# 19S proteasome loss regulates mitotic spindle assembly through a ubiquitin-independent degradation mechanism

**DOI:** 10.1016/j.celrep.2025.116041

**Published:** 2025-07-23

**Authors:** Océane Marescal, Iain M. Cheeseman

**Affiliations:** 1 Whitehead Institute for Biomedical Research, Cambridge, MA 02142, USA; 2 Department of Biology, Massachusetts Institute of Technology, Cambridge, MA 02142, USA; 3 Lead contact

## Abstract

During regulated protein degradation, the 26S proteasome recognizes ubiquitinated substrates through its 19S particle and then degrades them in its 20S enzymatic core. Despite this close interdependency between proteasome subunits, we demonstrate that knockouts from different proteasome subcomplexes result in distinct cellular phenotypes. In particular, depletion of 19S PSMD lid proteins, but not that of other proteasome subunits, prevents bipolar spindle assembly during mitosis. Despite decreased ubiquitin-mediated protein degradation in PSMD knockouts, we find that the monopolar spindle phenotype is instead caused by the aberrant degradation of the kinesin motor protein KIF11. We show that KIF11 degradation occurs through the 20S proteasome in a ubiquitin-independent manner upon loss of 19S proteins and that the resulting alterations in spindle forces lead to the unique monopolar phenotype. Thus, the presence of the 19S particle ensures proper spindle formation by restraining ubiquitin-independent degradation.

## In brief

Marescal and Cheeseman show that depletion of proteasome 19S lid subunits, but not that of other proteasome components, uniquely leads to the formation of monopolar spindles during mitosis. This phenotype arises from the aberrant ubiquitin-independent degradation of the motor protein KIF11 by the 20S proteasome core upon 19S loss.

## Graphical Abstract

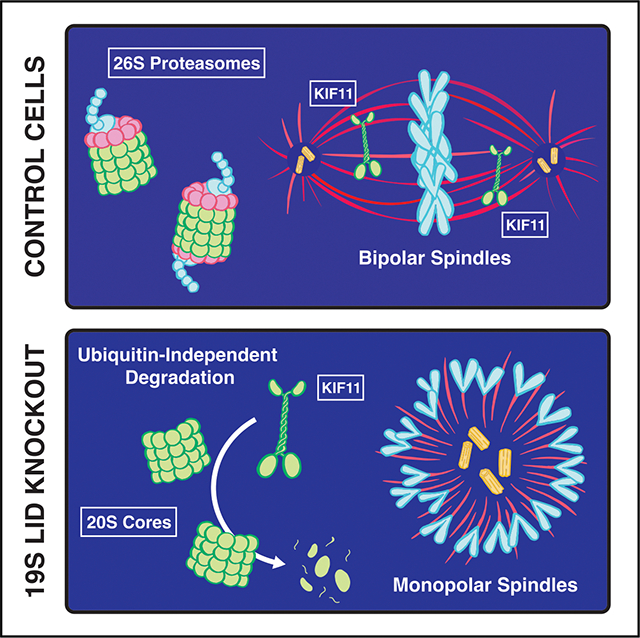

## INTRODUCTION

Proper formation of the mitotic spindle is crucial for cell division. The mitotic spindle is a microtubule-based structure that binds to chromosomes during mitosis and segregates them into two daughter cells.^1–4^ To achieve its function, the spindle must assemble into a bipolar configuration.^1–4^ Bipolar spindle formation begins with the duplication of centrioles during interphase to form two distinct microtubule-organizing centers called centrosomes.^1,3,5,6^ During mitosis, the centrosomes move to opposite ends of the cell to create two spindle poles from which microtubules emanate.^1,2^ Aberrant spindle assembly can lead to the formation of multipolar spindles with multiple spindle poles or monopolar spindles in which chromosomes surround a single pole.^1,5^ Such spindle assembly defects have deleterious consequences for the cell, resulting in mitotic arrest, cell death, or incorrect segregation of genetic material.^5,7^ Given the importance of the mitotic spindle in cell growth and disease, identifying the complete set of factors required for spindle assembly is critical.

**Figure 1.**
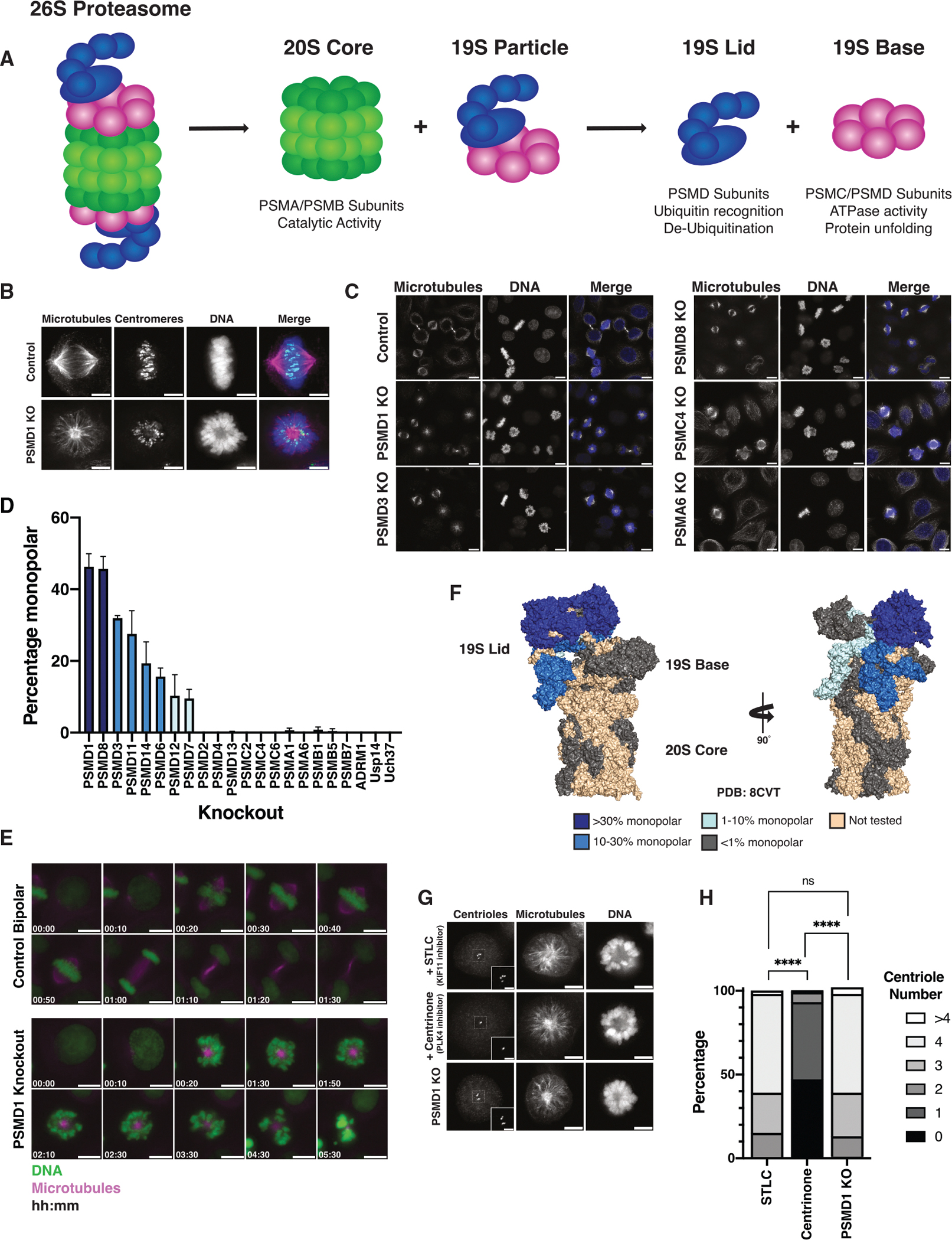
Depletion of a subset of 19S proteasome subunits induces monopolar spindles (A) Diagram depicting the 26S proteasome and its subcomplexes. (B) Representative immunofluorescence images of mitotic cells with a control bipolar spindle (top) or a monopolar spindle resulting from knockout of PSMD1 (bottom). Scale bars: 5 μm. Images were acquired on different days, and brightness is not scaled identically. (C) Representative immunofluorescence images of asynchronous control HeLa cells or HeLa cells with knockout of PSMD1, PSMD3, PSMD8, PSMC4, or PSMA6. Scale bars: 10 μm. Images were acquired on different days, and brightness is not scaled identically. (D) Graph showing the percentage of mitotic cells with monopolar spindles observed in cells with inducible CRISPR-Cas9 knockout of different proteasome components. Bars represent mean ± standard deviation of three or more replicates for each subunit. Between 56 and 234 mitotic cells (average 102) were quantified in each replicate. (E) Live-imaging stills of a PSMD1-inducible knockout cell entering mitosis or a control bipolar cell entering mitosis. Scale bars: 5 μm. Time units are h:min. (F) Structure of the 26S proteasome^16^ (PBD: 8CVT). Subunits are colored according to the percentage of monopolar cells observed in inducible CRISPR-Cas9 knockouts. (G) Representative images of monopolar mitotic cells from different conditions: cells treated with 10 μM STLC, cells treated with 200 nM centrinone,^17^ and inducible CRISPR-Cas9 PSMD1 knockout cells. Cells were stained for centrioles with a Centrin 2 antibody. Regions enlarged in insets are outlined with dashed squares. Image brightness is not identical. Scale bars for full-sized images: 5 μm. Scale bars for insets: 2 μm. (H) Quantification of centriole number under different conditions from (G). Bars show the percentage of cells in each centriole number category. The experiment was replicated 4 times, 36–51 cells were quantified for each condition for each replicate. *p* values were calculated with chi-square tests. ****p < 0.0001; ns, not significant.

The ubiquitin-proteasome system is a central player in cellular regulation and is the major pathway for regulated protein degradation.^8^ Proteins are marked for degradation by the covalent attachment of chains of ubiquitin to lysine residues, a post-translational-modification known as poly-ubiquitination that targets a protein to the 26S proteasome.^9,10^ The 26S proteasome is a large protein complex whose subunits can be grouped into three subcomplexes^8,11,12^ (Figure 1A). The 20S core particle, composed of PSMA and PSMB subunits, contains the proteasome’s catalytic activity.^8,11^ The 20S particle can be capped on either or both sides by the 19S regulatory particle, which can be further subdivided into the 19S base (PSMC/D subunits) and the 19S lid (PSMD subunits).^8,11^ The unfolding and translocation of protein substrates into the core is driven by the ATPase subunits of the 19S base, whereas the 19S lid is involved primarily in the recognition of ubiquitinated substrates and their de-ubiquitination.^8,10–12,13–15^

Ubiquitin-mediated degradation of specific substrates by the 26S proteasome is required for the cell cycle and for mitotic progression.^18–21^ For example, the degradation of cyclins by the ubiquitin pathway mediates transitions through each phase of the cell cycle,^22^ while anaphase onset during mitosis also relies on the ubiquitin-mediated degradation of specific protein components.^23,24^ Despite the well-studied role of the proteasome in cell cycle regulation, knowledge of its function in spindle formation remains limited. In addition, the proteasome’s function in the cell cycle beyond the canonical 26S complex, including the roles of individual proteasome subunits and the effects of ubiquitin-independent degradation, is poorly understood.

Here, based on the analysis of cellular phenotypes, we uncover the effects of proteasome depletion on mitotic bipolar spindle assembly. Using systematic knockout of proteasome components, we tested the effects of individual proteasome subunit depletion on cellular phenotype. Surprisingly, we find that the depletion of proteins from the 19S proteasome lid, but not that of other proteasome components, uniquely leads to the formation of monopolar spindles. Although 19S lid knockouts were deficient in ubiquitin-mediated degradation, we show that decreased degradation is not the cause of failed spindle formation. Instead, we reveal that this phenotype is caused by the aberrant ubiquitin-independent proteasomal degradation of a key spindle assembly factor, the kinesin motor protein KIF11, which occurs upon the loss of 19S lid subunits from the proteasome enzymatic core. Thus, the 19S lid restrains 20S proteasome degradation of specific cellular proteins so that, in the absence of this negative regulation, changes in ubiquitin-independent degradation lead to a failure in spindle assembly.

## RESULTS

### Depletion of a subset of 19S proteasome subunits induces monopolar spindles

To study the role of the proteasome in mitotic progression, we systematically generated individual CRISPR-Cas9-mediated knockouts of proteasome components from every proteasome subcomplex using a doxycycline-inducible knockout system in HeLa cells and visualized their phenotypes by microscopy^25^ (Table 1). This approach allowed us to dissect the individual contributions of different proteasome subunits to cellular phenotype, in contrast to studying the effects of the general inhibition of proteasomal degradation. We found that knockout of a subset of proteasome subunits resulted in a surprising mitotic phenotype: the formation of mitotically arrested cells with monopolar spindles, a spindle assembly defect in which cells have only a single spindle pole instead of the normal bipolar spindle structure (Figures 1B–1E, S1A, and S1B; Video S1).

Strikingly, only the knockout of PSMD subunits from the 19S particle, and not that of any other proteasomal component, resulted in monopolar spindle formation. These PSMD subunits are primarily structural components located within the lid of the 19S complex as well as the 19S base subunit PSMD1 (Figures 1D and 1F). By contrast, knockout of PSMA or PSMB subunits from the 20S core particle or PSMC ATPase subunits did not produce any monopolar mitotic cells (Figures 1C, 1D, and 1F). We also used RNAi to deplete proteasome subunits. Depletion of the 20S catalytic subunit PSMB7 by RNAi did not result in cells with monopolar spindles, whereas cells treated with small interfering RNAs (siRNAs) targeting the 19S subunit PSMD1 displayed a high percentage of monopolar cells (74%) (Figures S1C–S1E). The knockout or knockdown of PSMD1 in other cell types, including the non-small cell lung cancer cell line A549 as well as mouse (NIH 3T3), rabbit (LLC-RK), and dog (MDCK) cell lines also resulted in mitotically arrested cells with monopolar spindles (Figure S1F). Together, these results indicate that bipolar spindle assembly requires proper proteasome function and that only a specific subregion of the proteasome, the 19S lid, plays a unique role in regulating spindle bipolarity. Thus, different proteasomal subunits have distinct roles in the regulation of mitosis and cellular morphology, consistent with recent work highlighting the possibility of increased separation of function of proteasome subcomplexes.^29–33^

### Centriole duplication is not disrupted in PSMD knockout cells

As the proteasome had not been implicated previously in mitotic spindle formation, we sought to understand the basis for the monopolar phenotype. We first considered other factors whose loss would lead to similar spindle defects, such as genes involved in centriole duplication.^1,29^ Knockout or inhibition of known centriole replication factors, such as PLK4, which can be chemically inhibited by the small molecule centrinone, or SAS6^34–37^ resulted in monopolar spindles with reduced centriole numbers (Figures 1G, 1H, S1G, and S1H). In contrast, PSMD1 knockout cells had normal centriole numbers (Figures 1G, 1H, S1G, and S1H). Cells treated with *S*-trityl-L-cysteine (STLC), a kinesin-5 inhibitor that induces monopolar spindles without affecting centriole replication, also did not have reduced centriole numbers^7^ (Figures 1G and 1H). Thus, the knockout of PSMD subunits induces monopolar spindle formation without disrupting centriole duplication.

### Decrease in ubiquitin-dependent degradation is not the cause of monopolarity

**Figure 2.**
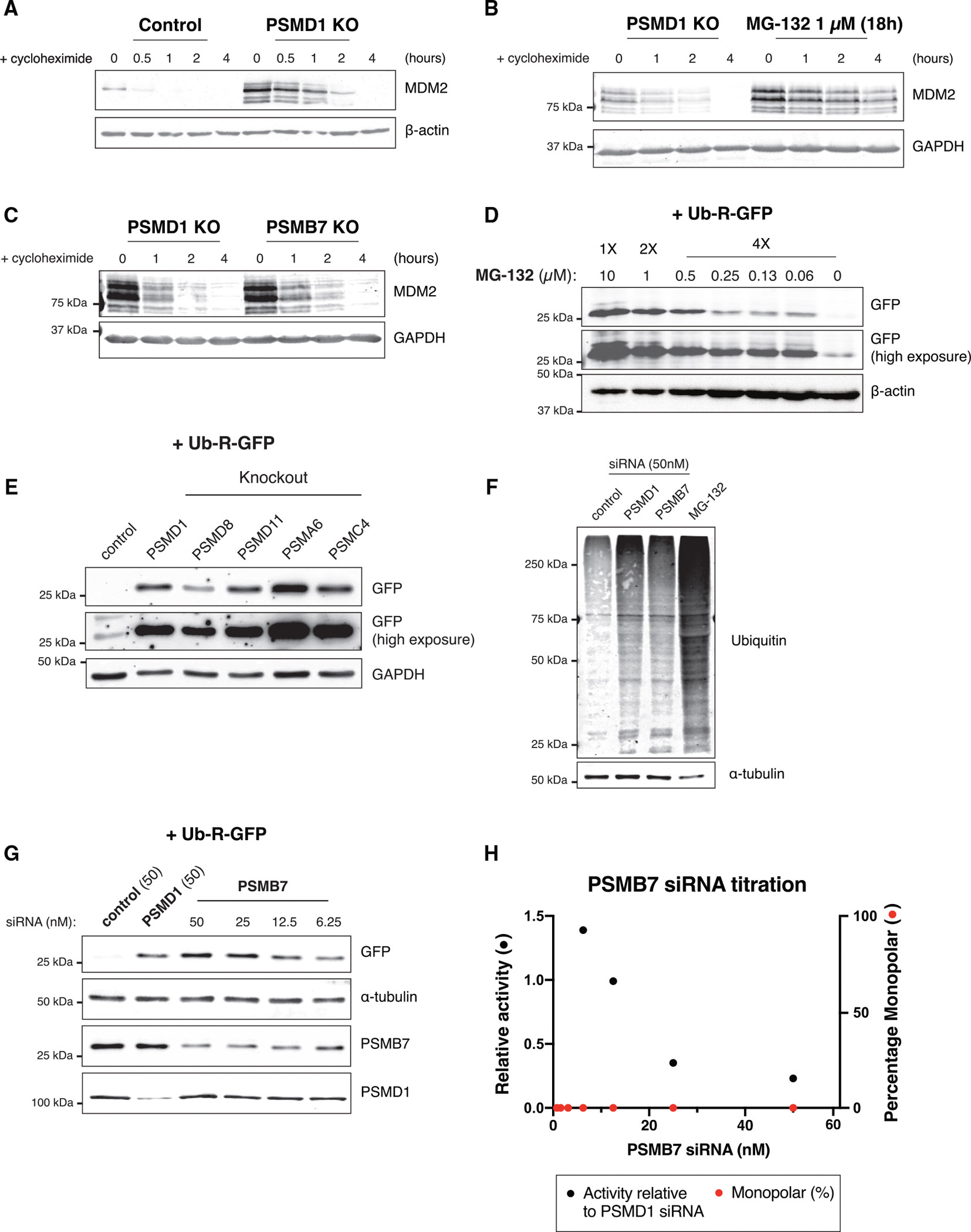
Decrease in ubiquitin-dependent degradation is not the cause of monopolarity (A) Western blot of control or inducible PSMD1 knockout cells treated with 50 μg/mL of cycloheximide for the indicated amount of time. The blot was incubated with an MDM2 antibody. Due to lane limitations, no ladder was run with the samples. However, the blot was run under the same conditions as previously with characteristic single banding patterns (see B and C and Figures 5D, 6A, S2A, S2B, S5C, and S6A–S6C). (B) Western blot of inducible PSMD1 knockout cells or cells treated with 1 μM MG-132 treated with 50 μg/mL of cycloheximide for the indicated amount of time. The blot was incubated with an MDM2 antibody. (C) Western blot of inducible PSMD1 and PSMB7 knockout cells treated with 50 μg/mL of cycloheximide for the indicated amount of time. The blot was incubated with an MDM2 antibody. (D) Western blot of cells transfected with the Ub-R-GFP construct treated with the indicated concentrations of MG-132. The blot was incubated with a GFP antibody. Lower and higher exposure times are shown. (E) Western blot of control cells or proteasome subunit knockout cells transfected with the Ub-R-GFP construct. Knockouts were generated by transducing HeLa cells with a lentivirus containing the mCherry-expressing gene knockout plasmids for the indicated genes. Cells were then sorted for mCherry-positive cells by fluorescence-activated cell sorting. The blot was incubated with a GFP antibody. Lower and higher exposure times are shown. (F) Western blot of cells treated with 50 nM control siRNAs, 50 nM PSMD1 siRNAs, 50 nM PSMB7 siRNAs, or 10 μM MG-132. The blot was incubated with a ubiquitin antibody. (G) Western blot of cells treated with 50 nM control siRNAs, 50 nM PSMD1 siRNAs, and the indicated concentrations of PSMB7 siRNAs, transfected with the Ub-R-GFP construct. The blot was incubated with a GFP, PSMB7, or PSMD1 antibody. (H) Graph showing the amount of proteasome activity in cells treated with different concentrations of PSMB7 siRNAs relative to the activity in cells treated with 50 nM PSMD1 siRNAs (black dots) and the percentage of monopolar cells observed by immunofluorescence in cells treated with different concentrations of PSMB7 siRNAs (red dots). The *y* axis indicates the relative amount of proteasome activity or percentage of monopolar cells. Activity values were calculated by quantifying the intensity of GFP bands from (G). Each measurement for the PSMB7 knockdown cells was divided by the value measured for PSMD1 knockdown cells. The percentage of monopolar cells was zero for all concentrations over three replicates of the experiment.

One of the primary roles of the 19S particle is to recognize ubiquitinated substrates for proteasomal degradation.^8,10–12,13–15^ Given this role, we next tested the effects of PSMD subunit depletion on the degradation of ubiquitinated proteins. We conducted a cycloheximide chase assay and used the levels of MDM2, a ubiquitinated protein with high turnover,^38,39^ as a reporter for ubiquitin-mediated protein degradation. In control cells, the inhibition of new protein synthesis led to the rapid loss of MDM2^39^ (Figure 2A). By contrast, cells treated with the proteasome inhibitor MG-132 maintained high levels of MDM2 over time (Figure 2B). Similarly, knockout of the 19S subunits PSMD1, PSMD8, and PSMD11, as well as the core and base subunits PSMB7 and PSMC4, each showed decreased degradation of MDM2 (Figures 2A–2C, S2A, and S2B).

We also assessed cellular degradation by measuring the stabilization of an exogenously expressed degron-tagged GFP construct^40^ (Ub-R-GFP). Cells treated with MG-132 showed an increase in the levels of Ub-R-GFP, indicating decreased degradation of the ubiquitinated construct (Figure 2D). Depletion of the 20S core components PSMA6 or PSMB7 or of the ATPase PSMC4 also stabilized Ub-R-GFP (Figures 2E, 2G, and S2D; Table S1). Similarly, knockouts of the 19S subunits PSMD1, PSMD8, and PSMD11 each stabilized the Ub-R-GFP fusion (Figures 2E, 2G, and S2C; Table S1). We also observed a global increase in the total levels of ubiquitin-conjugated proteins in cells treated with PSMD1 siRNAs, PSMB7 siRNAs, or MG-132 (Figure 2F). Thus, although only the knockout of 19S lid (PSMD) subunits induces monopolarity, depletion of components from all subregions of the proteasome decreases ubiquitin-mediated degradation, as expected from the previously established function of these proteasome components. Consistent with this loss in regulated protein degradation, the depletion of either 19S or 20S proteasome components, including PSMD subunits, resulted in eventual cell death, as proteasomal activity is essential for cell viability (Figures S2E–S2H; Video S1).

Although the depletion of PSMD subunits reduced ubiquitin-mediated degradation, in some cases this reduction was less potent than that observed for 20S core subunit-depleted cells. Thus, we considered whether a partial reduction of proteasome activity in PSMD knockouts could be the cause of monopolar spindle formation. To test this, we titrated siRNAs against PSMB7, a 20S core component, to induce varying levels of cellular protein degradation. Lower concentrations of PSMB7 siRNAs resulted in higher proteasome activity, as measured by the degradation of Ub-R-GFP (Figures 2G and 2H). However, we did not observe monopolar cells at any concentration of PSMB7 siRNAs, even at concentrations where proteasome activity was equivalent to that found in cells treated with PSMD1 siRNAs (12.5 nM PSMB7 siRNA) (Figures 2G and 2H). Thus, the knockout of components from any subcomplex of the proteasome decreases ubiquitin-mediated degradation, but the relative decrease in cellular degradation does not account for the cellular monopolar spindle phenotype observed in PSMD-depleted cells.

### Eg5/KIF11 is uniquely lost in PSMD-depleted cells

**Figure 3.**
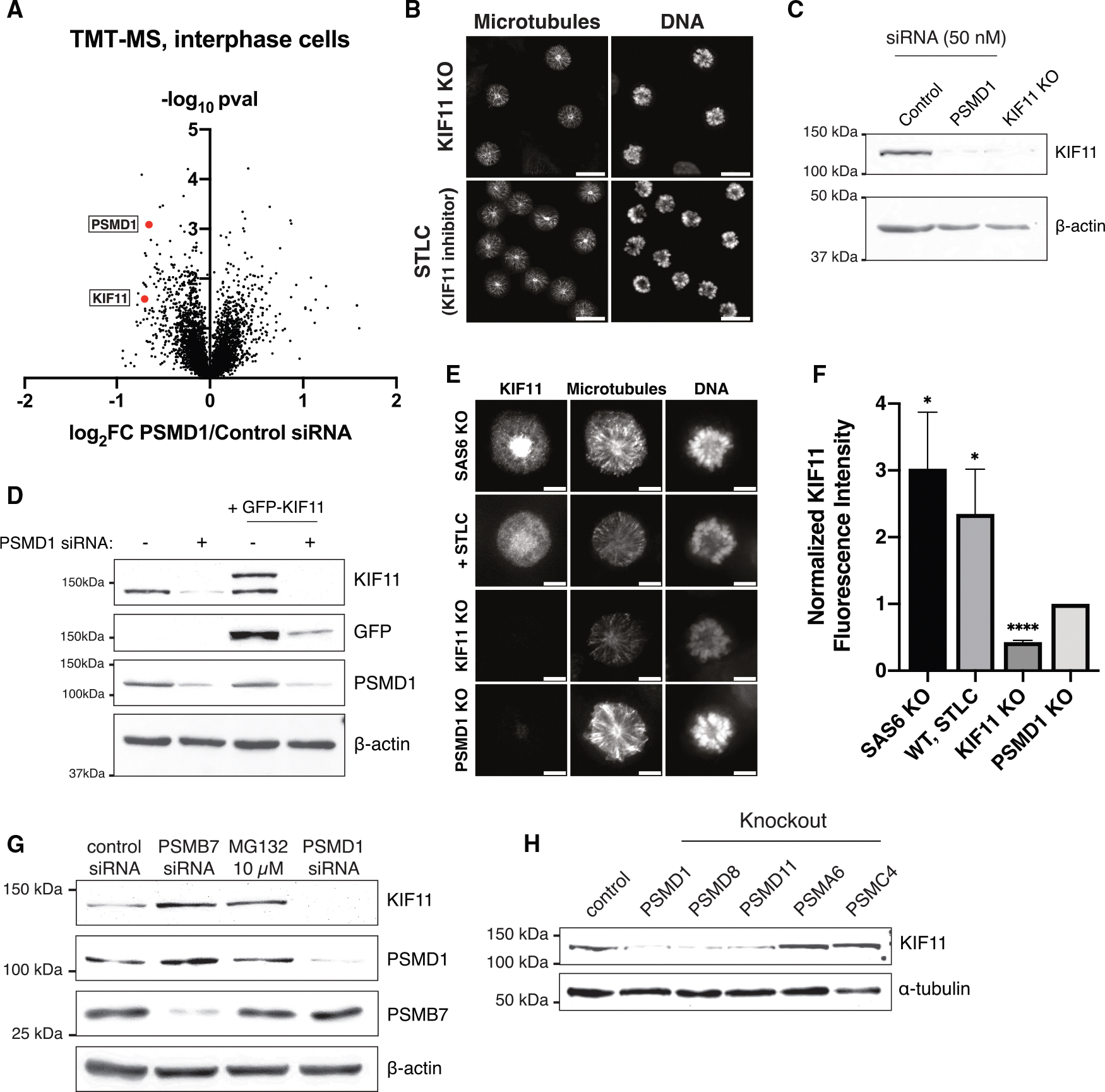
Eg5/KIF11 is uniquely lost in PSMD-depleted cells (A) Volcano plot comparing protein abundance as measured by quantitative MS in cells treated with 50 nM PSMD1 siRNAs and cells treated with 50 nM control siRNAs. PSMD1 and KIF11 are highlighted in red. (B) Representative immunofluorescence images of KIF11-inducible knockout cells and cells treated with 10 μM *S*-trityl-L-cysteine (STLC). Scale bars: 20 μm. Cells were fixed and stained on different days, and brightness is not scaled identically. (C) Western blot of cells treated with control siRNAs, cells treated with PSMD1 siRNAs, and inducible KIF11 knockout cells. The blot was incubated with a KIF11 antibody. (D) Western blots of a control or GFP-KIF11-expressing cell line treated with 50 nM control (−) or PSMD1 (+) siRNAs. The blots were incubated with KIF11, GFP, and PSMD1 antibodies. Separate blots were used for each antibody with the same samples and same amounts loaded. (E) Representative immunofluorescent images of monopolar mitotic cells showing KIF11 levels under different conditions: SAS6-inducible knockout cells (positive control), control cells treated with 10 μM STLC, KIF11-inducible knockout cells, and PSMD1 inducible knockout cells. Scale bar: 5 μm. (F) Quantification of KIF11 fluorescence intensity in monopolar mitotic cells under different conditions. Fluorescence intensity values were normalized to levels in PSMD1 knockout cells. Bars represent mean ± standard deviation. *p* values were calculated with two-tailed Welch’s t tests comparing each condition to PSMD1 knockout: *p* = 0.0275 for STLC, *p* = 0.0173 for SAS6 knockout, and *p* < 0.0001 for KIF11 knockout. *p < 0.05, ****p < 0.0001. The experiment was replicated 4 times; 29–92 cells were quantified for each condition for each replicate. (G) Western blots of cells treated with either 50 nM control siRNAs, 50 nM PSMB7 siRNAs, 50 nM PSMD1 siRNAs, or 10 μM MG132. Blots were incubated with a KIF11 antibody, PSMD1 antibody, and PSMB7 antibody. Separate blots were used for each antibody with the same samples and same amounts loaded. (H) Western blot of cells transduced with a lentivirus containing the mCherry-expressing gene knockout plasmids for PSMD1, PSMD8, PSMD11, PSMA6, or PSMC4. Cells were sorted for mCherry. Control cells are untransduced parental cells. The blot was incubated with a KIF11 antibody.

PSMD knockout cells have a potent monopolar spindle phenotype that could not be explained by defects in centriole duplication (Figures 1G, 1H, S1G, and S1H). To determine what other factors were responsible for the observed monopolarity in PSMD-depleted cells, we compared global changes in protein abundance between cells treated with either control siRNAs or PSMD1 siRNAs using tandem mass tag (TMT) quantitative mass spectrometry (Figures 3A and S3A; Tables S2 and S3). Given the decrease in ubiquitin-mediated degradation in PSMD1-depleted cells (Figures 2A, 2E, 2G, and S2C), we first searched for an upregulated protein whose lack of degradation could lead to monopolar spindle formation. Although several proteins that undergo ubiquitin-mediated degradation were increased in PSMD1 knockdown cells^22,41–47^ (Tables S2 and S3), none were relevant to spindle assembly. However, in both interphase and mitotic cells, one of the most significantly downregulated factors in PSMD1-depleted cells was the protein Eg5/KIF11 (Figures 3A and S3A; Tables S2 and S3). KIF11 is a kinesin-5 motor protein that separates spindle poles during spindle assembly.^1–3,48,49^ Loss of KIF11 activity through depletion or chemical inhibition results in potent monopolar spindle formation^1,2,48–50^ (Figure 3B).

Consistent with the reduction in KIF11 protein levels observed by mass spectrometry, we observed the loss of the endogenous KIF11 protein in PSMD1 knockdown cells using western blotting (Figure 3C). Similarly, we found that exogenously expressed GFP-tagged KIF11 was also lost in PSMD1 knockdown cells (Figure 3D). In addition, we detected decreased KIF11 spindle localization in PSMD1, PSMD3, PSMD8, and PSMD11 knockout cells based on immunofluorescence against the endogenous protein (Figures 3E, 3F, S3B, and S3C). Immunofluorescence in rabbit LLC-RK cells also showed reduced KIF11 spindle levels upon PSMD1 knockdown (Figures S3D and S3E). In contrast, depletion of PSMA6, PSMB7, or PSMC4 or the addition of MG-132 did not decrease KIF11 levels (Figures 3G and 3H) and instead resulted in an accumulation of KIF11. Thus, although depletion of other proteasome subunits or addition of a chemical proteasome inhibitor increases KIF11 levels, knockout of PSMD subunits leads to a unique loss of KIF11 protein.

### Loss of KIF11 contributes to the monopolar spindle phenotype in PSMD-depleted cells

**Figure 4.**
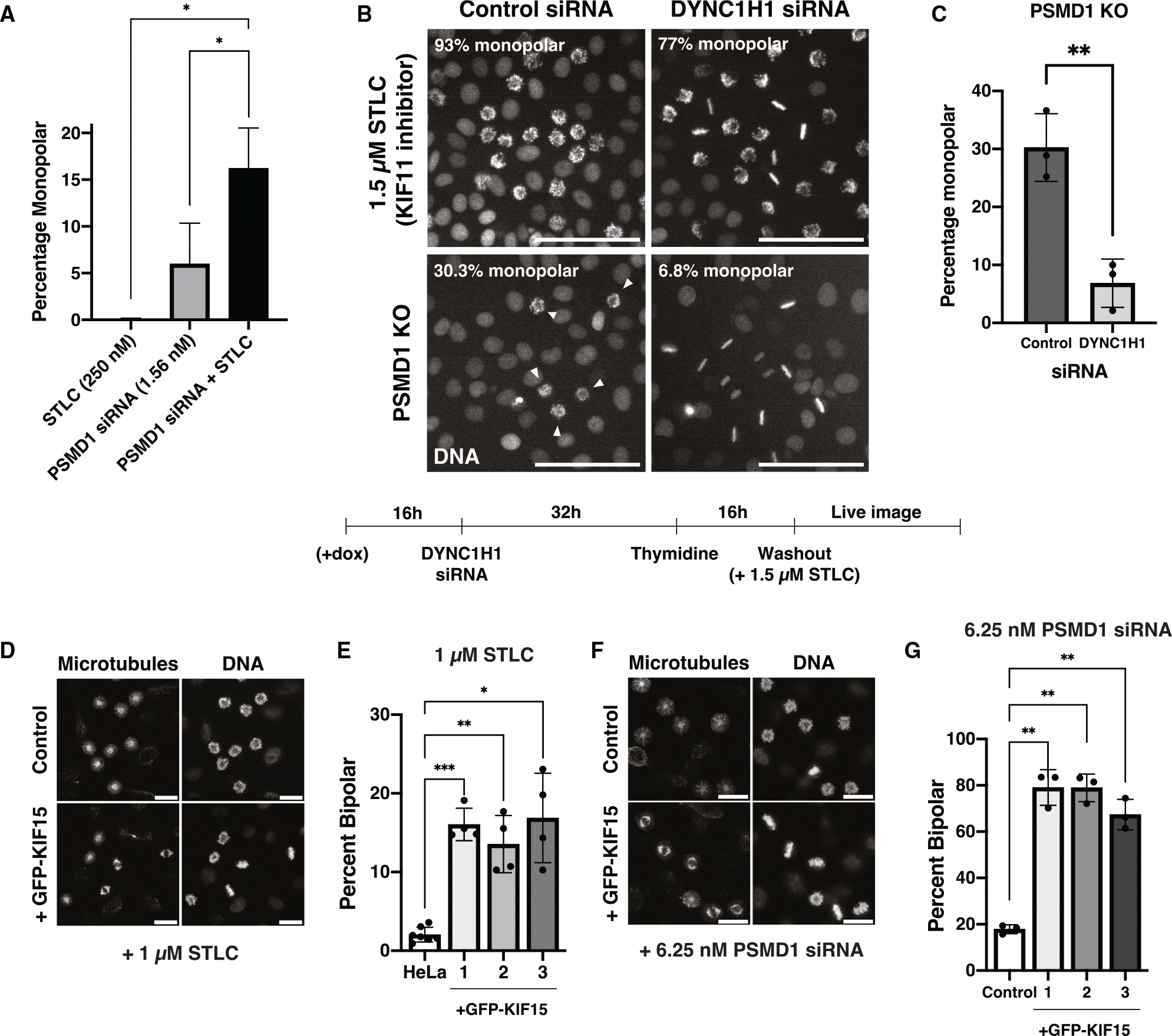
Loss of KIF11 contributes to the monopolar spindle phenotype in PSMD-depleted cells (A) Quantification of the live-imaging synergy experiment. The bar graph plots the percentage of cells entering mitosis with monopolar spindles in cells treated with a low dose (250 nM) of STLC alone, a low dose (1.56 nM) of PSMD1 siRNAs alone, or both 250 nM STLC and 1.56 nM PSMD1 siRNAs together. *p* values were calculated with two-tailed Welch’s t tests; *p* = 0.023 for STLC vs. siRNA + STLC, and *p* = 0.045 for PSMD1 siRNA vs. siRNA + STLC. *p < 0.05. The experiment was replicated 3 times, and between 1,090 and 2,527 mitotic cells were counted for each condition for each replicate. (B) Representative stills of live-imaging experiments. Top: Cells were treated with either 50 nM control siRNAs or 50 nM dynein heavy chain (DYNC1H1) siRNAs and then with 1.5 μM of the KIF11 inhibitor STLC. The experiment was conducted once, as these results have been studied previously.^51^ Bottom: doxycycline was added to cells to induce knockout of PSMD1. Cells were then treated with either 50 nM control siRNAs or 50 nM DYNC1H1 siRNAs. White arrows point toward monopolar cells. Scale bars: 100 μm. A diagram of the experimental timeline is displayed underneath the images. (C) Quantification of the dynein rescue experiment from (B). The bar graph shows the percentage of cells entering mitosis with monopolar spindles in PSMD1 knockout cells treated with 50 nM of either control or DYNC1H1 siRNAs. *p* values were calculated with two-tailed Welch’s t tests; *p* = 0.0064. **p < 0.01. The experiment was conducted 3 times, and between 1,215 and 1,521 mitotic cells were counted for each condition in each replicate. (D) Representative immunofluorescence images of either control or GFP-KIF15-expressing cells treated with 1 μM STLC. Scale bars: 20 μm. Image brightness is not scaled identically. (E) Bar graph showing the percentage of mitotic cells with bipolar spindles in control HeLa cells or 3 different clones of a GFP-KIF15-overexpressing cell line treated with 1 μM STLC. *p* values were calculated with two-tailed Welch’s t tests; *p* = 0.0003, 0.0068, and 0.013 between HeLa and clone 1, 2, and 3, respectively. ***p < 0.001, **p < 0.01, *p < 0.05. Experiments were replicated 3 times, and between 208 and 555 mitotic cells were counted for each condition in each replicate. (F) Representative immunofluorescence images of either control or GFP-KIF15-expressing cells treated with 6.25 nM PSMD1 siRNAs. Scale bars: 20 μm. (G) Bar graph showing the percentage of mitotic cells with bipolar spindles in control HeLa cells or 3 different clones of a GFP-KIF15-overexpressing cell line treated with 6.25 nM PSMD1 siRNAs. *p* values were calculated with two-tailed Welch’s t tests; *p* = 0.0035, 0.0015, and 0.0034 between HeLa and clone 1, 2, and 3, respectively. **p < 0.01. Experiments were replicated 3 times, and between 121 and 318 mitotic cells were counted for each condition in each replicate.

We next evaluated whether the loss of KIF11 is sufficient to explain spindle monopolarity in PSMD-depleted cells. Ectopically expressed GFP-KIF11 was also degraded in PSMD1 knockdown cells (Figure 3D) and thus failed to restore bipolar spindle formation (Figures 6E and 6F). We therefore used alternate methods to evaluate the contributions of KIF11. We predicted that a reduction of KIF11 levels in PSMD-depleted cells would sensitize these cells to further KIF11 inhibition. We therefore analyzed the synergistic phenotypes between partial PSMD1 depletion using RNAi and partial inhibition of KIF11 activity using the chemical inhibitor STLC.^7^ Although treating cells with low concentrations of STLC or low concentrations of PSMD1 siRNAs alone did not result in substantial monopolarity (Figures 4A and S4A–S4C), simultaneously treating cells with both STLC and PSMD1 siRNAs resulted in an almost 3-fold increase in monopolar cells (Figures 4A and S4C).

KIF11 separates spindle poles by producing outward force on the spindle.^52^ Thus, if the loss of KIF11 contributes to spindle monopolarity in PSMD-depleted cells, then either decreasing inward force or restoring outward force on the spindle should rescue spindle bipolarity. Prior work found that the depletion of the motor protein dynein rescues bipolar spindle formation in cells with reduced KIF11 activity by eliminating opposing inward force on the spindle^2,51^ (Figure 4B, top). To test whether dynein depletion can also rescue monopolarity induced by loss of PSMD1, we treated PSMD1 knockout cells with siRNAs against dynein heavy chain (DYNC1H1) (Figure S4D). PSMD1 knockout cells treated with DYNC1H1 siRNAs formed significantly fewer monopolar spindles than those treated with control siRNAs (Figures 4B, bottom, and 4C). Similarly, siRNAs against the dynein-binding protein Lis1 also rescued spindle bipolarity in PSMD1 knockouts (Figure S4E).

Reciprocally, restoring outward spindle forces in cells with low KIF11 levels is predicted to rescue bipolar spindle formation. Previous work found that the motor protein KIF15 (kinesin-12) acts in overlapping pathways with KIF11 to push spindle poles apart.^53^ The overexpression of KIF15 can rescue the loss of KIF11 activity^2,50^ so that GFP-KIF15 overexpression restored spindle bipolarity in cells treated with STLC^50^ (Figures 4D, 4E, S4F, and S4G). Similarly, in cells treated with PSMD1 siRNAs, we found that cells overexpressing GFP-KIF15 had a significantly higher percentage of bipolar cells than control cells (Figures 4F and 4G).

Thus, KIF15 overexpression can rescue the effects of PSMD1 depletion despite there being no difference in KIF15 protein levels in PSMD1 knockdown cells (Figure S4H). Thus, experimental manipulations known to rescue the loss of KIF11 activity by either increasing the amount of outward force (KIF15 overexpression) or decreasing the amount of inward force on the spindle (dynein heavy chain or Lis1 RNAi) rescue PSMD subunit depletion. Together, these data suggest that the loss of KIF11 in PSMD-depleted cells contributes to the monopolar phenotype generated upon proteasome lid subunit depletion.

### KIF11 loss occurs through proteasomal degradation

We next sought to uncover the mechanism by which KIF11 is lost in PSMD-depleted cells. In addition to reduced KIF11 protein levels, we found that the levels of the endogenous KIF11 mRNA were decreased upon PSMD1 knockdown (Figure S5A). However, KIF11 mRNA levels also decreased significantly in PSMB7 knockdown cells, which do not have decreased KIF11 protein levels (Figures S5A and 3G). We also found similar rates of KIF11 accumulation over time between PSMD1 knockdown and control knockdown cells treated with MG-132, indicating similar rates of protein production (Figures S5C and S5D). In addition, GFP-KIF11 protein expressed from an ectopic promoter was also lost in PSMD1 knockdown cells (Figure 3D) despite there being no difference in the change in mRNA levels upon PSMD1 knockdown of the GFP-KIF11 construct relative to that of GFP alone (Figures S5B and S5E). These experiments suggest that KIF11 loss occurs primarily at the protein level, although the decrease in KIF11 mRNA in PSMD-depleted cells could also play a role in lowering levels of the endogenous protein.

**Figure 5.**
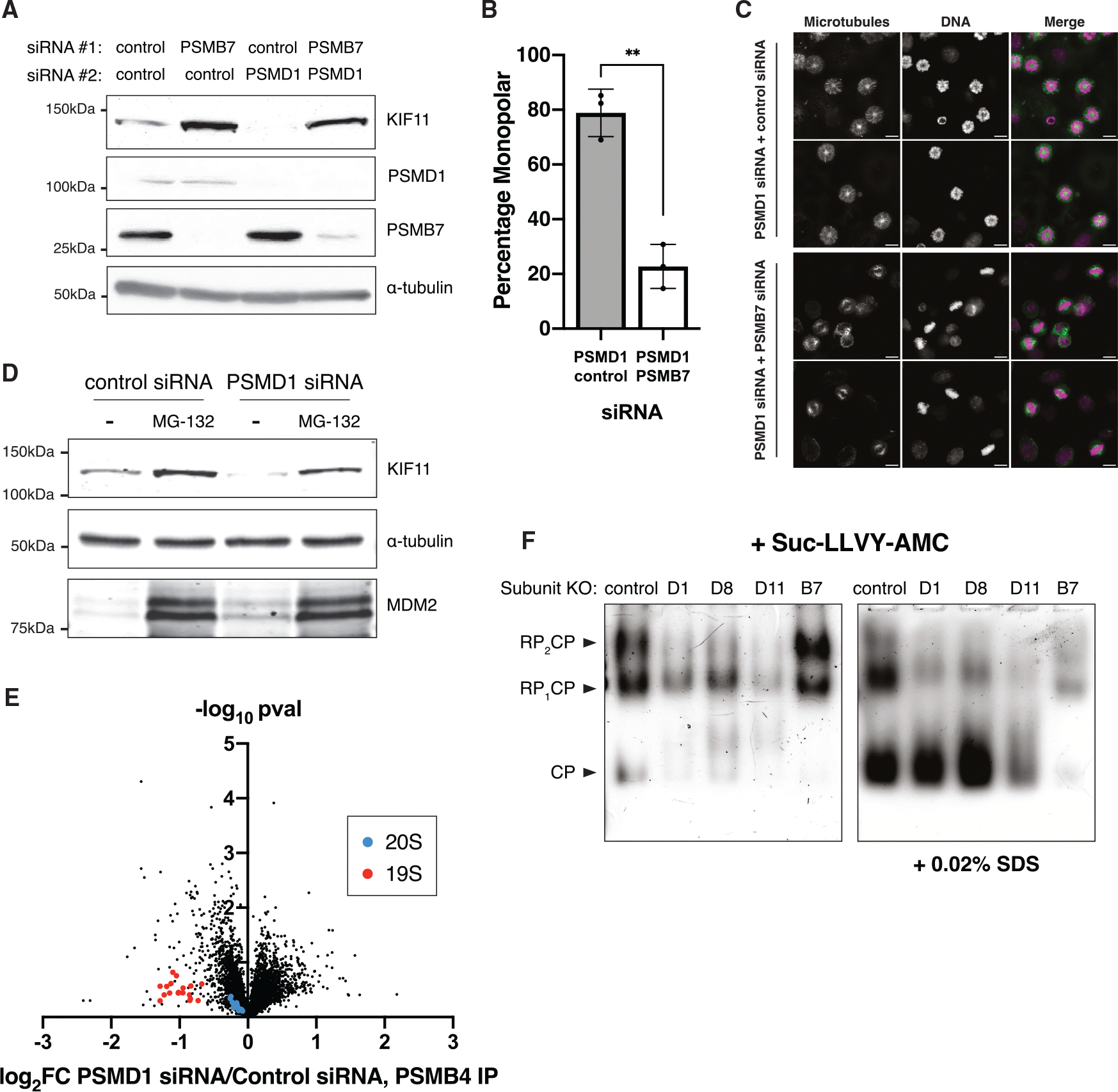
KIF11 loss is mediated by proteasomal degradation (A) Western blots of double knockdown cells. Cells were treated with 50 nM siRNA #1 for 72 h and siRNA #2 for 48 h before collection. Blots were incubated with KIF11, PSMD1, and PSMB7 antibodies. Separate blots were used with the same samples and same amounts loaded. (B) Quantification of the immunofluorescence experiment from PSMD1/PSMB7 double-knockdown cells. The bar graph shows the percentage of mitotic cells with monopolar spindles for cells treated with both 12.5 nM PSMD1 and 50 nM control or 50 nM PSMB7 siRNAs. The *p* value was calculated with two-tailed Welch’s t tests; *p* = 0.0012. **p < 0.01. Between 545 and 2184 (average, 1,307) mitotic cells were counted for each condition, and the experiment was replicated 3 times. (C) Representative immunofluorescence images for the experiment in (B). Scale bars: 10 μm. Image brightness is not scaled the same. (D) Western blots of cells treated with 50 nM of the indicated siRNAs with or without 10 μM MG-132. Blots were incubated with KIF11 and MDM2 antibodies. Separate blots were used with the same samples and same amounts loaded. (E) Volcano plot comparing protein abundance as measured by quantitative MS of GFP IP from GFP-PSMB4 cells treated with 50 nM PSMD1 siRNAs and cells treated with 50 nM control siRNAs. 19S components and 20S components are highlighted in red and blue, respectively. (F) Native gel followed the by Suc-LLVY-AMC assay using different proteasome-inducible knockout cell lines. Bands corresponding to double-capped (RP_2_CP), single-capped (RP_1_CP), or uncapped 20S proteasomes (CP) are indicated. Addition of 0.02% SDS opens the 20S proteasome substrate channel and allows for detection of 20S proteasome activity.

To test whether loss of KIF11 protein is mediated by the proteasome, we simultaneously depleted both PSMD1 and the 20S catalytic core component PSMB7 using RNAi. Although treating cells with PSMD1 siRNAs alone led to an almost complete loss of KIF11 protein, simultaneously depleting both PSMD1 and PSMB7 prevented the degradation of KIF11 (Figure 5A) and led to a 4-fold decrease in the fraction of monopolar cells (Figures 5B and 5C). Similarly, PSMA6 knockout cells treated with PSMD1 siRNAs retained KIF11 protein and showed rescue of bipolar spindle formation (Figures S5F–S5H). Finally, addition of the chemical proteasome inhibitor MG-132 impeded the degradation of KIF11 (Figures 5D, S5C, and S5D). Thus, genetic or chemical inhibition of proteasome activity rescues KIF11 protein levels and bipolar spindle formation in PSMD-depleted cells, suggesting that KIF11 is degraded by the proteasome despite an overall decrease in ubiquitin-mediated degradation (Figure 2).

### KIF11 degradation is ubiquitin independent

Depletion of 19S lid subunits is predicted to prevent formation of a capped 26S proteasome but could potentially leave the 20S catalytic core intact.^54–57^ We therefore measured the association of 19S subunits with the 20S core using immunoprecipitation followed by quantitative mass spectrometry (IP-MS) (Figure 5E; Table S4). IP of the core subunit PSMB4 in PSMD1 knockdown cells pulled down substantially less of each 19S subunit when compared to control knockdown cells, suggesting a dissociation of the proteasome 19S particle from the core upon PSMD1 knockdown (Figure 5E). Conversely, the interaction of PSMB4 with other core subunits was unaffected (Figure 5E; Table S4). We also used native gels to measure the ratio of different proteasome complexes and their catalytic activity (Figure 5F). Native gels separate whole proteasome complexes by size without compromising their activity, allowing for the *in vitro* cleavage of the fluorescent substrate Suc-LLVY-AMC^58–60^ (Suc-Leu-Leu-Val-Tyr-AMC) to detect which populations of proteasome complexes are capable of substrate cleavage. We observed a decrease in the 26:20S proteasome ratio in PSMD knockout cells but found that the accumulated 20S cores retained their catalytic cleavage activity (Figure 5F), consistent with decreased ubiquitin-mediated degradation but preservation of 20S core proteasome activity.

**Figure 6.**
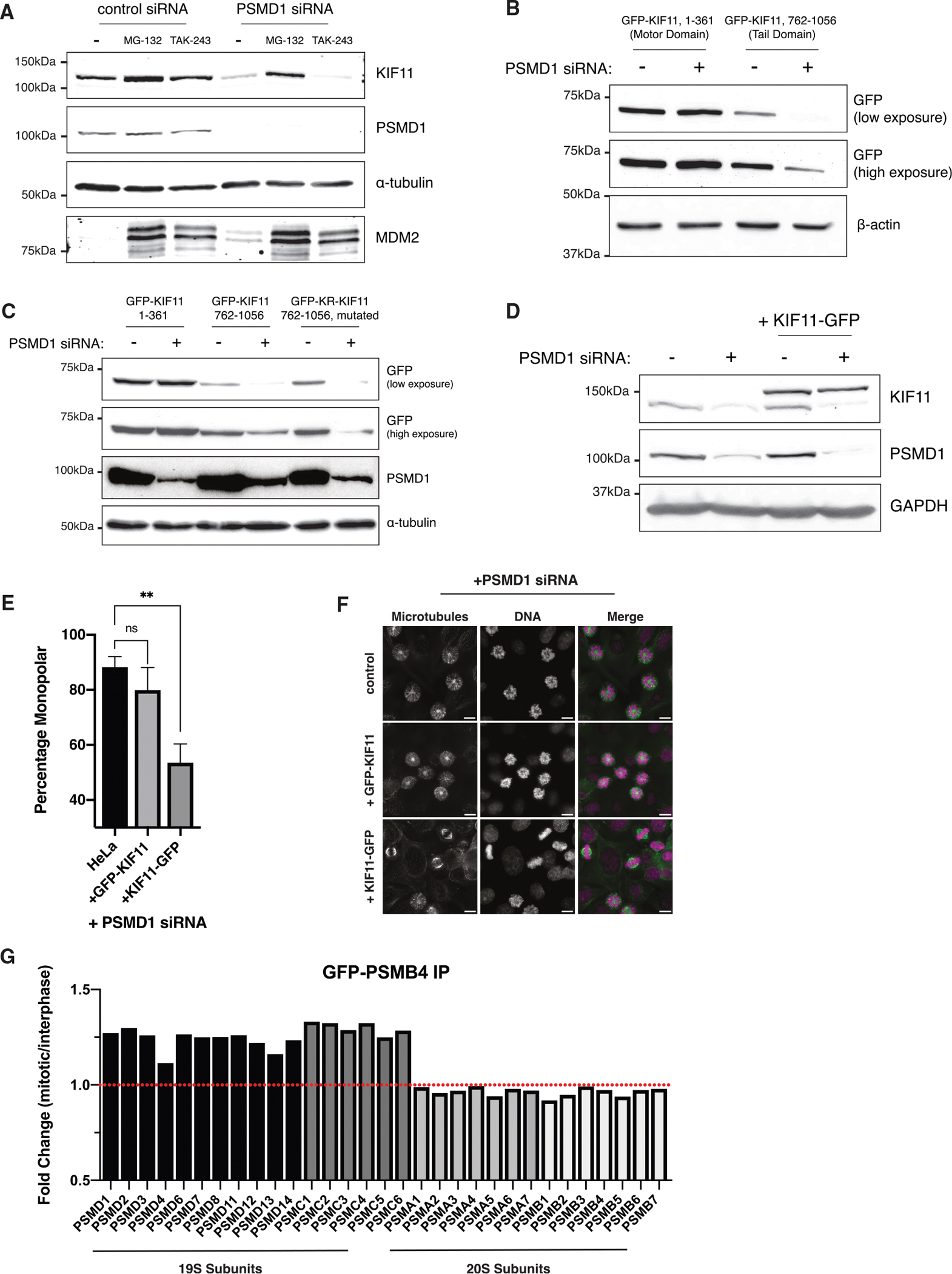
Proteasomal degradation of KIF11 is ubiquitin independent and mediated by its unstructured C terminus (A) Western blots of cells treated with 50 nM siRNAs and the indicated compounds. MG-132 was used at 10 μM and TAK-243 at 300 nM. Blots were incubated with KIF11, PSMD1, and MDM2 antibodies. Separate blots were used with the same samples and same amounts loaded. (B) Western blot of cell lines expressing the indicated GFP-KIF11 truncation constructs treated with 50 nM control (−) or PSMD1 (+) siRNAs. The blot was incubated with a GFP antibody. Lower and higher exposure times are shown. (C) Western blot of cell lines expressing the indicated GFP-KIF11 truncation constructs treated with 50 nM control (−) or PSMD1 (+) siRNAs. The blot was incubated with GFP and PSMD1 antibodies. Lower and higher exposure times are shown. (D) Western blot of control cells or a cell line expressing a C-terminally tagged KIF11-GFP construct. Cells were treated with either 50 nM control (−) or PSMD1 (+) siRNAs. The blot was incubated with KIF11 and PSMD1 antibodies. (E) Quantification of the immunofluorescence experiment for cells expressing KIF11 constructs. The bar graph shows the percentage of mitotic cells with monopolar spindles for parental HeLa cells, a cell line expressing N-terminally tagged GFP-KIF11, and a cell line expressing C-terminally tagged KIF11-GFP treated with 12.5 nM PSMD1 siRNAs. *p* values were calculated with two-tailed Welch’s t tests; *p* = 0.0039 and *p* = 0.3703 between HeLa and KIF11-GFP and GFP-KIF11, respectively. ns = not significant, **p < 0.01. (F) Representative immunofluorescence images from (E). Scale bar: 10 μm. (G) Bar graph showing fold change of protein abundance pulled down in mitotic compared to asynchronous cells, as measured by quantitative MS. GFP IP was performed on GFP-PSMB4 cell lines in mitotic (STLC-arrested) and asynchronous cells. The bar graph shows the abundance ratios of mitotic/asynchronous values for proteasome 19S and 20S subunits.

Although poly-ubiquitination and subsequent degradation by the 26S proteasome is the most frequently used cellular degradation pathway, some substrates have also been shown be degraded in a ubiquitin-independent manner.^61–63^ As the 19S lid is the ubiquitin-recognizing component of the 26S proteasome and dissociates from the 20S core upon PSMD depletion, we hypothesized that the degradation of KIF11 in lid-depleted cells could be proteasome dependent but ubiquitin independent. To test whether degradation of KIF11 is ubiquitin independent, we treated PSMD1 knockdown cells with either MG-132 to inhibit proteasome catalytic activity or TAK-243 to inhibit ubiquitination. TAK-243 (MLN7243) is an inhibitor of the ubiquitin-activating E1 enzyme UBA1 and disrupts ubiquitination upstream of proteasome activity.^64^ Although addition of either MG-132 or TAK-243 decreased degradation of the ubiquitin-dependent substrate MDM2, only the addition of MG-132 rescued KIF11 levels in PSMD1-depleted cells (Figure 6A). This ubiquitin-independent degradation behavior was also observed in cells treated with PSMD8 siRNAs and is conserved in rabbit LLC-RK cells (Figures S6A and S6B). Thus, TAK-243-treated PSMD knockdown cells continue to degrade KIF11 even in the absence of E1 activity and ubiquitination. In contrast to PSMD knockdown cells, control cells treated with either MG-132 or TAK243 both accumulated KIF11 (Figure S6C), consistent with previous work showing ubiquitin-mediated degradation of KIF11 upon mitotic exit.^65,66^

### The unstructured C terminus of KIF11 mediates its degradation

To determine the region of KIF11 responsible for its degradation, we created truncations of the protein and assessed their degradation. In contrast to the full-length protein, the N-terminal motor domain of KIF11 (amino acids 1–361) did not show reduced protein levels upon PSMD1 knockdown (Figures 6B and S6D). However, the depletion of PSMD1 did induce the loss of the KIF11 C-terminal tail domain (amino acids 762–1056) (Figures 6B and S6D). Because ubiquitination of substrate proteins occurs through the covalent attachment of ubiquitin to lysine residues, we also created a lysine-to-arginine mutant of the C-terminal tail domain of KIF11 (GFP-KR-KIF11, 762–1056). Although the lysine-less KIF11 fragment would be incapable of being ubiquitinated, it was still degraded upon PSMD1 knockdown (Figures 6C; S6D). Thus, the degradation of KIF11 depends on its C-terminal domain but not on its ubiquitination.

Previous cases of ubiquitin-independent degradation have been found to require the presence of an unstructured region to initiate translocation into the proteasome.^32,67–69^ Structural models of KIF11 predict the presence of an ~130-amino-acid intrinsically disordered region at the C terminus of the protein (Figures S6E and S6F). Because our C-terminal construct was degraded in our truncation experiments (Figures 6B and 6C), we tested whether this exposed disordered region was essential for the ubiquitin-independent degradation of KIF11. To do so, we tagged KIF11 with GFP at its C terminus (KIF11-GFP). Although an N-terminally tagged GFP-KIF11, whose C terminus remains available, was degraded efficiently upon addition of PSMD1 or PSMD8 siRNAs (Figure 3D), we found that the C-terminally tagged KIF11-GFP was not (Figures 6D, S6G, and S6H).

Having uncovered the mechanism by which KIF11 is degraded and a means to prevent KIF11 loss, we next sought to test whether KIF11 levels alone are sufficient to rescue spindle formation in 19S-depleted cells. To do so, we knocked down PSMD1 in cells expressing either N-terminally tagged GFP-KIF11 or non-degradable C-terminally tagged KIF11-GFP. Although GFP-KIF11 did not rescue spindle formation in PSMD1 siRNA-treated cells, we observed an increase in spindle bipolarity in cells expressing KIF11-GFP (Figures 6E and 6F). We observed the same GFP-KIF11 and KIF11-GFP behavior in cells treated with PSMD8 siRNA (Figures S6H and S6I). Thus, blocking KIF11’s free disordered C-terminal region with GFP prevented the protein’s degradation, and the consequent rescue of KIF11 levels is sufficient to rescue monopolar spindles induced by PSMD subunit depletion.

From these data, we conclude that the loss of 19S PSMD components triggers the ubiquitin-independent proteasomal degradation of KIF11, which is dependent on its disordered C terminus.

### Proteasome lid-base association increases in mitotic cells

As the loss of 19S lid components had unique effects on cellular phenotype and proteasome degradation behavior, we sought to identify conditions under which the proteasome 26:20S ratio could be altered. In particular, we considered whether association of the proteasome lid with the 20S core could vary between different cell cycle stages. To test this, we immunoprecipitated GFP-PSMB4, a 20S subunit, from asynchronous and mitotic cells and analyzed the abundance of co-immunoprecipitated proteins using quantitative MS (Table S5). This approach allowed us to compare the amount of 19S proteins associated with the 20S core between interphase and mitosis. Interestingly, we observed an increase in the association of all 19S particle subunits with the proteasome core during mitosis (Figure 6G), while the total amount of these proteins remained stable between cell cycle stages (Figure S7A). We also observed a 2-fold increase in KIF11 abundance during mitosis (Figure S7B). Although the degradation of KIF11 upon mitotic exit is known to be ubiquitin dependent,^65,66^ the increased 26:20S proteasome ratio in mitotic cells could also contribute to maintaining KIF11 levels high during this cell stage, when its function is needed most.

## DISCUSSION

The canonical pathway for protein degradation is the ubiquitination of target proteins followed by their degradation by the 26S proteasome. Here, we show that the depletion of different proteasome subunits can give rise to vastly different cellular behaviors, highlighting the capacity for independent functions of the 19S and 20S particles outside of the canonical 26S complex. We find that the knockout of 19S PSMD proteasome components causes a failure in spindle pole separation during mitosis, resulting in the formation of aberrant monopolar spindles. However, despite functioning in the same complex, other subunits of the proteasome from the 19S base or the 20S core do not induce a similar monopolar phenotype upon depletion. These results strengthen the growing belief that the proteasome may function not only as a 26S entity but also as alternative or partial complexes that may each have their own independent cellular roles.^30,32,33,61–63,67,70,71^

Our results find that decreases in the 26:20S proteasome ratio downregulate the levels of the kinesin-5 motor protein KIF11. As a primary factor in mitotic bipolar spindle assembly, even small changes in KIF11 abundance can have drastic effects on cellular growth and viability.^72,73^ Thus, maintaining appropriate levels of KIF11 is essential for the survival and proper division of a cell, and the control of proteasome component abundance could be an important mechanism for regulating KIF11 levels and proper spindle formation under different conditions.

Here, we identified the C terminus of KIF11 as an essential region for its degradation upon PSMD subunit depletion. Recent work defined specific C-terminal motifs that can drive ubiquitin-independent degradation, including the presence of alanine, cysteine, or valine at the end of a protein.^61^ However, KIF11 does not contain these C-degron sequences. Instead, the protein has a highly unstructured C terminus (Figures S6E and S6F), which is known to render proteins susceptible to 20S degradation.^32,67–69^ Indeed, blocking this unstructured region through the addition of a structured C-terminal GFP tag abrogated KIF11 degradation upon PSMD subunit depletion (Figure 6D). Interestingly, structural predictions of KIF11 from other species suggest that the presence of this C-terminal disordered region is conserved in eukaryotes from fungi to vertebrates (Figure S7C). We found that the ubiquitin-independent degradation of KIF11 in PSMD-depleted cells also occurred in rabbit cells (Figure S6A). Thus, the conservation of an unstructured C terminus could perhaps indicate the preservation of KIF11 degradation through a ubiquitin-independent mechanism across species.

The proteasome 19S particle has important functions in substrate unfolding, ubiquitin recognition, and deubiquitination.^10,13–15^ Our data also suggests an additional role for the 19S particle as a negative regulator of proteasome degradation activity by the 20S core. We demonstrate that, in the absence of the 19S lid, degradation by the 20S particle can have deleterious consequences for the cell. This suggests that the 19S lid plays a role not only in mediating ubiquitin-dependent degradation by the 26S proteasome but also in preventing ubiquitin-independent degradation of a subset of substrates by the 20S core. In the case of KIF11, the 19S lid may also play a role in directly stabilizing the protein, in addition to preventing its degradation. Although IP experiments of both the lid and KIF11 did not reveal any stable direct physical interaction between the 19S particle and KIF11 (Figures S7D–S7G; Table S6), it is possible that a transient or indirect interaction could mediate a stabilizing effect. For example, the 19S could directly stabilize a protein that further modifies KIF11. The 19S particle also contains multiple intrinsic and associated deubiquitinases (DUBs), which have been shown previously to mediate certain 19S moonlighting functions.^33^ The knockout of the intrinsic DUB PSMD14 resulted in monopolar spindle formation (Figure 1D). However, knockout of associated DUBs, Usp14 and Uch37, as well as the chemical inhibition of all DUB activities using the small molecules capzimin and b-AP15,^74,75^ did not cause monopolar spindles (Figure 1D; data not shown).

The negative regulation of substrate degradation by the 19S lid may also be important in the context of the cell cycle. The knockout of 19S components had adverse effects on cell cycle progression due to the ubiquitin-independent degradation of KIF11. In a more endogenous context, we also found differences in 19S association with the 20S core between mitosis and interphase. Specifically, we observed increased association of 19S subunits with the 20S core during mitosis (Figures 6G; Table S5), which would be predicted to reduce ubiquitin-independent degradation of KIF11 in mitotic cells. Although the degradation of KIF11 during mitotic exit is known to be ubiquitin-dependent,^65,66^ it is also possible that the fluctuations in 26:20S ratios throughout the cell cycle may play a parallel role in mediating KIF11 degradation and stabilization. It remains to be seen whether a combination of ubiquitin-dependent and ubiquitin-independent degradation contributes to the regulation KIF11 levels.

In unperturbed cells, only half of the proteasome complexes are found in lidded forms, with the other half existing as uncapped 20S complexes.^76,77^ Despite this, the occurrence of monopolar spindles in a normal cell population is rare. How normal cells prevent the ectopic degradation of KIF11 by free 20S cores is still an open question. One solution to this would be the increased association of 19S particles to the 20S core specifically during mitosis, when the cell requires KIF11 function (Figure 6G). Another option could be the compartmentalization of proteasomes away from KIF11. For example, proteasomes are known to localize to the nucleus during interphase, whereas KIF11 is mostly cytoplasmic.^78,79^ Other possibilities include the existence of an alternative negative regulator of 20S activity in normal cells or that of a regulatory component whose association upon loss of the 19S lid could control proteasomal substrate recognition.

Finally, we found that PSMD-depleted cells arrest strongly in mitosis. These results suggest that PSMD subunits may be fruitful targets for cancer therapeutics, as cancerous cells are highly proliferative and would be susceptible to selective death through a mitotic arrest mechanism. Indeed, certain cancer cell types are reliant on high 26:20S ratios for their continued proliferation, and high PSMD1 levels are correlated with a poor survival prognosis in patients with oropharyngeal squamous cell carcinoma.^80,81^ Thus, the development of therapeutic strategies that either deplete 19S lid subunits or dissociate proteasome complexes to decrease the 26:20S ratio could lead to selective arrest and killing of highly proliferative cancer cells and improve patient outcomes.

### Limitations of the study

We show that the knockout of several different 19S components, including PSMD1, PSMD3, PSMD8, and PSMD11, results in the loss of KIF11. However, further experiments that investigate the KIF11 phenotype were conducted only with PSMD1 and PSMD8 depletion. We assume that the results would be similar across all other PSMD subunit knockouts. The degradation of KIF11 and spindle monopolarity are observed in the context of 19S depletion. However, it is still uncertain whether KIF11 degradation by 20S cores also occurs in unperturbed cells.

## RESOURCE AVAILABILITY

### Lead contact

Requests for further information, resources, and reagents should be directed to and will be fulfilled by the lead contact, Iain Cheeseman (icheese@wi.mit.edu).

### Materials availability

All reagents generated in this study will be made available by the lead contact upon request.

### Data and code availability

- All data reported in this manuscript will be shared by the lead contact upon request.
- MS proteomics data have been deposited to the ProteomeXchange Consortium via the PRIDE (Proteomics Identification Database) partner repository and are publicly available as of the date of publication. Accession numbers are listed in the key resources table.
- Full western blot image data have been deposited into Mendeley data and are publicly available as of the date of publication. The DOI is listed in the key resources table.
- This paper does not report original code.
- Any additional information required to reanalyze the data reported in this paper is available from the lead contact upon request.

## STAR★METHODS

### EXPERIMENTAL MODEL AND STUDY PARTICIPANT DETAILS

#### Cell lines

HeLa, A549, 3T3, LLC-RK1, and MDCK cell lines were cultured in Dulbecco’s modified Eagle medium (DMEM) supplemented with 10% fetal bovine serum (FBS), 100 U/ml penicillin and streptomycin, and 2mM L-glutamine at 37°C with 5% CO_2_. Doxycycline-inducible cell lines were cultured in medium containing tetracycline-free FBS. HeLa cells are female in origin, isolated from the cervical carcinoma of a 31-year-old human patient. A549 cells were isolated from a 58-year-old male with lung cancer. The NIH3T3cell line was derived from a male mouse embryo. LLC-RK1 cells were derived from the kidney of New Zealand white rabbits, sex unspecified. MDCK were isolated from normal kidney tissue of adult female cocker spaniel. Cell lines were tested monthly for mycoplasma contamination.

### METHOD DETAILS

#### Cell culture and reagents

For CRISPR-Cas9 inducible knockout cell lines, *sp*Cas9 expression was induced with 1 μg/mL doxycycline hyclate (Sigma-Aldrich). Doxycycline media was renewed every 24 h. Other drugs used on human cells were cycloheximide (50 μg/mL; Sigma-Aldrich), MG-132 (concentrations denoted in figures or figure legends; Enzo Life Sciences), taxol (1 μM; Invitrogen), thymidine (2 mM, Sigma-Aldrich), STLC (10 μM, unless otherwise noted in figure or figure legends; Sigma-Aldrich), and centrinone (200 nM, Oegema-Desai lab).^17^ Transfections of Ub-R-GFP (Addgene Plasmid #11939) were done using Effectene Transfection Reagent (Qiagen).

#### Cell line generation

Inducible CRISPR-Cas9 HeLa and H2B-mCherry HeLa cell lines were generated from previously described parental cell lines.^25^ sgRNAs were cloned into the sgOpti plasmid (puro-resistant, Addgene plasmid #85681) and introduced into the parental cell line by lentiviral transduction, followed by selection with 0.5 μg/mL puromycin (Gibco). sgRNA guide sequences can be found in Table S7.

GFP-KIF15 cell lines were generated by transfection using X-tremeGENE-9 (Roche) of GFP-KIF15 plasmid (gift from Patricia Wadsworth)^83^ followed by selection with 800 μg/mL Geneticin (G418 Sulfate, Thermo Fisher). Monoclonal cell lines were isolated by fluorescence-activated cell sorting and selected based on characteristic GFP localization by immunofluorescence.

GFP-KIF11 cell lines (including wild-type KIF11, KtoR KIF11, and KIF11 fragments) and the GFP control cell line were generated by lentiviral transduction. Lentivirus was made by transfection with Xtremegene-9 (Roche) of GFP or GFP-KIF11-containing lentivirus plasmid, VSV-G envelope plasmid, and psPAX2 (Addgene plasmid #12260) packaging plasmid into HEK-293T cells. GFP-positive cells were then sorted by fluorescence-activated cell sorting.

#### Western blot

For western blot experiments, cells were harvested and flash frozen in liquid nitrogen to be stored at −80°C or immediately lysed on ice for 25 min in urea lysis buffer (50 mM Tris pH 7.5, 150 mM NaCl, 0.5% NP-40, 0.1% sodium dodecyl sulfate (SDS), 6.5 M Urea, 1X Complete EDTA-free protease inhibitor cocktail (Roche), 1 mM phenylmethylsulfonyl fluoride (PMSF)). Centrifugation was used to remove cellular debris. Protein concentrations were measured using Bradford reagent (Bio-Rad) to normalize loading. Lysates were mixed with Laemmli sample buffer and 2-mercaptoethanol and heated at 95°C for 5 min. Samples were loaded onto 8, 10, or 12% acrylamide gels for SDS-PAGE and transferred to nitrocellulose or Polyvinylidene Fluoride (PVDF) membrane (Cytiva). Membranes were blocked for 1 h in blocking buffer (2.5% milk in Tris-Buffered Saline (TBS) + 0.1% Tween 20). Primary antibodies were diluted in 0.25% milk in TBS +0.1% Tween 20 and added to the membrane for 2 h at room temperature or overnight at 4°C. Membranes were washed with TBS +0.1% Tween 20 and then incubated in either HRP-conjugated secondary antibodies (GE Healthcare; Digital) or in IRDye secondary antibodies (LI-COR) for one hour at room temperature. After washing in TBS +0.1% Tween 20, membranes were either incubated in clarity-enhanced chemiluminescence substrate (Bio-Rad) and imaged using the KwikQuant Imager (Kindle Biosciences) or imaged using the Odyssey CLx Imager (LI-COR). Quantitative analysis of bands (Figures S5C and S5D) was performed in LI-COR Image Studio Software. β-actin, GAPDH, or α-tubulin are used as a loading controls. Antibodies are listed in Table S8.

#### Immunofluorescence

For immunofluorescence experiments, cells were plated on poly-L-lysine (Sigma-Aldrich)-coated coverslips. Following fixation with either Formaldehyde or Methanol, coverslips were washed with phosphate-buffered saline (PBS) + 0.1% Triton X-100 (PBS-Tx) and blocked in blocking buffer (20 mM Tris-HCl, 150 mM NaCl, 0.1% Triton X-100, 3% bovine serum albumin, 0.1% NaN_3_, pH 7.5) for 30 min or overnight. Primary antibodies were diluted in blocking buffer and added to coverslips for 1 h or overnight. Cells were washed with 0.1% PBS-Tx. Cy2-, Cy3-, or Cy5-conjugated secondary antibodies (Jackson ImmunoResearch Laboratories) were diluted in blocking buffer and added to coverslips for 1 h. Cells were washed with 0.1% PBS-Tx. 1 μg/mL Hoechst-33342 (Sigma-Aldrich) diluted in 0.1% PBS-Tx was added for 10 min to stain for DNA. Coverslips were mounted onto slides using ProLong Gold antifade reagent (Invitrogen). Images were taken with a DeltaVision Ultra High-Resolution microscope and analyzed with Fiji (ImageJ, NIH). A list of antibodies and their dilutions can be found in Table S8.

#### Live-cell imaging

For live-cell imaging, H2B-mCherry tagged cells were seeded onto 12-well glass-bottom plates (Cellvis, P12–1.5P). 1 h prior to imaging, the media was changed to CO_2_-independent media (Gibco) supplemented with 10% FBS, 100 U/ml penicillin and streptomycin, and 2 mM L-glutamine, with or without 1 μM SiR-tubulin for visualizing microtubules. Imaging was conducted at 37°C on a DeltaVision Ultra High-Resolution microscope (40X) or with a Nikon eclipse microscope (20X). Images were analyzed with Fiji (ImageJ, NIH).

#### Analysis of CRISPR-Cas9 cutting efficiency by TIDE

To analyze Cas9-based genome cutting efficiency, cell pellets from control and edited cells were resuspend in genomic DNA lysis buffer (100 mM Tris pH 8.0, 5 mM EDTA, 200 mM NaCl, 0.2% SDS, proteinase K to a final concentration of 0.2 mg/mL) and incubated overnight at 55°C. DNA was then precipitated the following day by addition of equal volume of isopropanol, then washed with 75% ethanol and resuspended in buffer (10 mM Tris-HCl pH 8.0, 0.1mM EDTA) overnight at 55°C in a shaking incubator. PCR was run using the isolated genomic DNA and the corresponding primers for each gene (Table S7). PCR products were run on 1–2% agarose gels, gel extracted, and tested by Sanger sequencing (Azenta). Trace files were used for quantitative assessment of genome editing by TIDE (Tracking of Indels by Decomposition, https://tide.nki.nl/).

#### siRNA treatment

Custom siRNAs against PSMD1 (pool: 5′-CAAAGGAUGCAGUACGGAA, 5′-AGACCAUACUGGAGUCGAA, 5′-GAUUGGAAGGCA UCGUAAA, 5′- CAUGGGAACUGCACGUCAA, or individual: 5′-AGACCAUACUGGAGUCGAA), PSMD8 (pool: 5′-GCUACUACUUUG AUUACAA, 5′-AAUUCUGGCCCGUGACAUA, 5′- GAGCUCAACUUCUUGCCAA, 5′- CAGUGUCCCUGGAGCAAUA), PSMB7 (pool: 5′-CCGCAGGAAUGCCGUCUUG, 5′-GUAUCAAGGUUACAUUGGU, 5′-GGUCUAUAAGGAUGGCAUA, 5′- GAAGAUAAGUUUAGG CCAG), DYNC1H1 (pool: 5′-GAUCAAACAUGACGGAAUU, 5′-CAGAACAUCUCACCGGAUA, 5′-GAAAUCAACUUGCCAGAUA, 5′- G CAAGAAUGUCGCUAAAUU), and LIS1 (pool: 5′-CAAUUAAGGUGUGGGAUUA, 5′-UGAACUAAAUCGAGCUAUA, 5′-GGAGUGCCGUUGAUUGUGU, 5′- UGACAAGACCCUACGCGUA), and a non-targeting control pool (D-001206–13) were obtained from Dharmacon. siRNAs were used at a final concentration of 50 nM, unless otherwise stated in the text or figure legend. siRNAs and Lipofectamine RNAiMAX (Invitrogen) were mixed at equal volume in Opti-MEM Reduced Serum Medium (ThermoFisher), vortexed, and allowed to incubate for 20 min before adding to cells. For double siRNA experiments, final concentration of both siRNAs was 50 nM for both with double the volume of Lipofectamine RNAiMAX. Media was changed between 8 and 24 h later back to full-serum media.

#### RNA isolation, reverse transcription, and qPCR

To isolate RNA, 400 μL of TRIzol RNA isolation reagent (ThermoFisher) was added to cells in a 6-well plate. Lysed cells were then transferred to a 1.5 mL centrifuge tube, vortexed, and frozen at −80°C. After thawing samples, 120 μL of chloroform was added and tubes were vortexed vigorously for at least 30 s before centrifuging at 4°C. The aqueous phase was then transferred to a new tube with equal volume of chloroform, vortexed, and spun again at 4°C. Aqueous phase was transferred to a new tube with GlycoBlue Coprecipitant (Invitrogen), 5 M NaCl, and equal volume of isopropanol, and incubated on dry ice then centrifuged at 4°C. Pellets were washed with 75% Ethanol and resuspended in water. Reverse transcription was performed using Maxima First Strand cDNA Synthesis Kit for RT-qPCR (Thermo Scientific). For qPCR, cDNAs were diluted 1:20. 1 μM primers and 2X SYBR Green PCR Master Mix (Thermo Fischer Scientific) were mixed with cDNAs in 384-well plates with three technical replicates per cDNA sample and primer pair. Primer sequences can be found in Table S7.

#### Immunoprecipitation

Beads were prepared prior to immunoprecipitation. NHS Mag Sepharose magnetic beads (Cytiva) were washed once with 500 μL of ice-cold 1 mM HCl, then mixed with 50 μg of GFP nanobody and Coupling Buffer B (0.2 M NaHCO_3_, 0.5 M NaCl, pH 8.3) to a total volume of 300 μL and incubated at room temperature for 1 h. Beads were then washed with Blocking Buffer A (0.5 M ethanolamine, 0.5 M NaCl, pH 8.3) and Blocking Buffer B (0.1 M Sodium Acetate, 0.5 M NaCl, pH 4) and incubated in Blocking Buffer A for 15 min. After incubation, beads were washed again with Blocking Buffers A and B, then resuspended in 0.2 M ethanolamine, 0.2 M NaCl at 4°C.

Cells were harvested and resuspended in equal volume of 1X Lysis Buffer (50 mM HEPES, 1 mM EGTA, 1 mM MgCl_2_, 100 mM KCl, 10% glycerol) with 2% Triton X-100, Complete EDTA-free protease inhibitor cocktail (Roche), 1 mM phenylmehtylsulfonyl fluoride (PMSF), 0.4 mM Sodium Orthovanadate, 5 mM Sodium Fluoride, 20 mM Beta-glycerophosphate, and ATP regeneration system (50 μg/mL creatine kinase, 35 mM creatine phosphate, 5 mM ATP). Cells were lysed on ice for 30 min prior to centrifuging at 15,000 rpm for 30 min. After centrifuging, ATP regeneration system was added to the supernatant before mixing with GFP-nanobody-conjugated beads for 2 h at 4°C. After incubation, beads were washed with wash buffer (50 mM HEPES, 1 mM EGTA, 1 mM MgCl_2_, 100 mM KCl, 10% glycerol, 2% Triton X-100, ATP regeneration system, 1 mM DTT, 10 μg/mL leupeptin (Millipore), 10 μg/mL pepsin (Thermo Fisher Scientific), 10 μg/mL chymostatin (Millipore)), before elution with 0.1M Glycine pH 2.6. Eluted samples were mixed with 200 mM Tris pH 8.5 and 20% Trichloroacetic Acid (Fisher Bioreagents) overnight.

#### Quantitative mass spectrometry

For whole-cell quantitative proteomics, cells were treated with either 50 nM PSMD1 siRNAs or 50 nM control siRNAs for 48 h. For interphase cells, 2 mM Thymidine (Sigma-Aldrich) was added 24 h prior to harvesting to arrest cells in S phase. For mitotic cells, 1 μM taxol (Invitrogen) was added 24 h prior to harvesting to arrest cells in mitosis. Mitotic cells were isolated by mitotic shake off. Three biological replicates were collected for each condition. Cells were flash frozen in liquid nitrogen and stored at −80°C. To prepare protein extracts, cells were resuspended in RIPA buffer (225 mM NaCl, 1.5% Nonidet P-40 substitute, 0.75% Na Deoxycholate, 0.15% SDS, 75 mM Tris pH 8) with Complete EDTA-free protease inhibitor cocktail (Roche), 1 mM phenylmehtylsulfonyl fluoride (PMSF), 0.4 mM Sodium Orthovanadate, 5 mM Sodium Fluoride, and 20 mM Beta-glycerophosphate. Cells were lysed with Branson Digital Sonifier tip sonicator at 30% amplitude for 10 s once. Lysates were centrifuged at 20,000g for 30 min at 4°C. Supernatant was then mixed with 20% Trichloroacetic Acid (Fisher Bioreagents) overnight on ice to precipitate proteins. The next day, samples were centrifuged at 20,000g at 4°C. Pellets were washed two times with cold acetone and dried in an Eppendorf Vacufuge.

To prepare samples for mass spectrometry, pellets were first resuspended in SDS lysis buffer (5%, 50 mM TEAB pH 8.5) with 20 mM DTT and incubated at 95°C for 10 min. Samples were then incubated with 40 mM iodoacetamide (Sigma) for 30 min in the dark. 1.2% phosphoric acid was added to the sample, which was then run over S-Trap microcolumns (ProtiFi) and subjected to tryptic digest on the columns overnight (Promega). Digested peptides were then eluted, quantified with Quantitative Fluorometric Peptide Assay Kit (Pierce), and lyophilized. A more detailed protocol for S-Trap digestion and elution can be found in the ProtiFi S-trap micro kit protocol.

For TMT-based quantitative mass spectrometry, peptides from each sample were mixed with different TMT10plex labels at a 1:10 ratio by mass. Labeling occurred in 30% acetonitrile and 24.5 mM TEAB pH8.5 for 1 h at room temperature. After 1 h, the reaction was quenched with hydroxylamine for 15 min at room temperature. TMT-labeled samples were then pooled, lyophilized, and stored at −80°C. Next, samples were fractionated using Pierce High pH Reversed-Phase Peptide Fractionation kit (Pierce). Fractions were lyophilized and resuspended in 0.1% formic acid and analyzed by mass spectrometry with an Orbitrap Exploris 480. Protein identification and Tandem Mass Tag quantification was done in Proteome Discoverer 2.4 (Thermo Fisher Scientific).

The following conditions were used for TMT-MS experiments in this work. For Figure 3A: Cells were treated first with control siRNAs, media was then changed. 24 h later, cells were treated again with PSMD1 or control siRNAs for 48 h and then arrested in S phase with 2 mM thymidine prior to harvesting. Protein abundances were obtained using TMT-based (Tandem Mass Tag) quantitative mass spectrometry of three replicates for each condition. For Figure S3A: Cells were transduced with lentivirus with a guide targeting a control AAVS safe-harbor locus. Then they were treated with either PSMD1 or control siRNAs for 48 h and arrested in mitosis with 1 μM taxol prior to harvesting. Mitotic cells were specifically isolated from interphase cells using mitotic shake off. Protein abundances were obtained using TMT-based (Tandem Mass Tag) quantitative mass spectrometry of three replicates for PSMD1 RNAi condition and two replicates for control RNAi condition. For Figure 5E: Cells were treated with siRNAs for 48 h and with 2 mM Thymidine 24 h prior to harvesting. Protein abundances were obtained using TMT-based (Tandem Mass Tag) quantitative mass spectrometry of three replicates for PSMD1 RNAi condition and two replicates for control RNAi condition.

#### Native gel and Suc-LLVY-AMC assay

Cells were harvested and resuspended in TSDG buffer (10 mM Tris HCl pH 8, 1.1 mM MgCl_2_, 10 mM NaCl, 0.1 mM EDTA, 1 mM NaN_3_, 1 mM DTT, 2 mM ATP, 10% glycerol). Lysis was carried out through 7 sequential freeze-thaws in liquid nitrogen. Lysates were centrifuged for 30 min at 4°C and supernatant was collected. Samples were mixed with glycerol and run on a 3.5% acrylamide gel containing 0.5mM ATP in Native Running Buffer (90mM Tris Borate, 5mM MgCl_2_, 0.5mM EDTA, 0.5mM ATP) for 3 h at 100V.

For Suc-LLVY-AMC assay: Following running, native gel was incubated in Suc-LLVY-AMC reaction buffer (50mM Tris pH 7.5, 0.5 mM ATP, 0.5 mM DTT, 0.04 mM Suc-LLVY-AMC substrate (Fischer Scientific)) for 30 min at 37°C. For activation of 20S particles, gel was imaged in reaction buffer +0.02% SDS for 30 min at 37°C. Gels were then imaged with ChemiDoc Imaging System (Bio-Rad).

#### Flow cytometry analyses

For DNA content analysis, cells were first harvested and washed with phosphate-buffered saline (PBS). Cells were then resuspended in 1 mL of PBS before addition of 9 mL of ice-cold ethanol. After ethanol fixation, cells were centrifuged, ethanol was aspirated away, and cells were resuspended in PBS to rehydrate. Afterward, cells were centrifuged again and resuspended in blocking buffer (20 mM Tris-HCl, 150 mM NaCl, 0.1% Triton X-100, 3% bovine serum albumin, 0.1% NaN_3_, pH 7.5) for 30 min. After blocking, cells were resuspended in PBS containing 10 μg/mL RNase A and 20 μg/mL propidium iodide (PI, Invitrogen) and incubated for 30 min before analysis by flow cytometry.

For analysis of cell death, live cells were stained with either propidium iodide (PI, Invitrogen) or 4′, 6-diamidino-2-phenylindole (DAPI, EMD Millipore). For PI staining, PI was added to cells at 1μL/mL for 15 min, cells were then harvested and immediately analyzed by flow cytometry. For DAPI staining, cells were harvested, resuspended in PBS containing 0.2μg/ml DAPI, and immediately analyzed by flow cytometry.

PI or DAPI signal was measured on an LSRFortessa (BD Biosciences) flow cytometer. Results were analyzed with FlowJo software. Data was collected on at least 10,000 cells for each condition per experiment.

### QUANTIFICATION AND STATISTICAL ANALYSIS

Quantification of fluorescence intensity for immunofluorescence experiments was conducted on unprocessed, maximally projected images using FIJI/ImageJ. These images were acquired using the same microscope and acquisition settings on the same day, unless otherwise noted in the figure legends. Quantification of western blot band intensities was done using LICORbio Image Studio. Statistical analyses were performed using Prism (GraphPad Software). Details of statistical tests and sample sizes are provided in the figure legends.

## Supplementary Material

1

2

3

4

5

6

7

SUPPLEMENTAL INFORMATION

Supplemental information can be found online at https://doi.org/10.1016/j.celrep.2025.116041.

## Figures and Tables

**Table 1.** Percentage editing of inducible CRISPR-Cas9 knockouts

Percentage editing of inducible CRISPR-Cas9 knockouts		

Complex	Subunit	Percentage editing

19S Base	PSMD1^a^	40.5
	PSMD2	56.3
	PSMC2	48.6
	PSMC4	49.1
	PSMC6	29.0
19S Lid	PSMD3	34.3
	PSMD6	20.3
	PSMD7	27.4
	PSMD8	49.0
	PSMD11	44.3
	PSMD12	40.8
	PSMD13	27.5
	PSMD14	49.2
	ADRM1	55.4
20S Core	PSMA1	46.3
	PSMA6	46.3
	PSMB1	39.6
	PSMB5	49.1
	PSMB7	40.7

Shown is the genome cutting efficiency of proteasome subunit CRISPR-Cas9-inducible knockouts as measured by tracking of insertions or deletions by decomposition. Knockout cell lines were generated as described previously,^25^ and gene knockout was induced by addition of doxycycline 2–3 days prior to collecting genomic DNA.

a PSMD1 (Rpn2) is canonically referred to as a 19S base subunit. However, due to its structural association with 19S lid components and phenotypic similarity to other PSMD lid subunits,^16,26–28^ we chose to include PSMD1 in our general definition of “lid” subunits that induce monopolar phenotypes.

**KEY RESOURCES TABLE:** 

REAGENT or RESOURCE	SOURCE	IDENTIFIER

Antibodies		

α-tubulin	Sigma-Aldrich	AB_477593; RRID_AB_477593
Anti-centromere Antibodies (ACA)	Antibodies, Inc.	15-234-0001; RRID: AB_2939058
PSMD1	Abcam	ab140682; RRID: AB_3711219
PSMB7	Proteintech	30283-I-AP; RRID: AB_3086285
PSMD8	Proteintech	27504-I-AP; RRID: AB_3669605
PSMD6 (Proteasome 19S Rpn7)	Enzo	BML-PW8225-0100; RRID: AB_10541630
β-actin (HRP conjugated)	Santa Cruz Biotechnology	sc-47778 HRP; RRID: AB_626632
MDM2	Cell Signaling Technology	D1V2Z; RRID: AB_2784534
GFP	Roche	11814460001; RRID: AB_390913
GAPDH	Santa Cruz Biotechnology	sc-47724; RRID: AB_627678
Eg5/KIF11	Abcam	ab181981; RRID: AB_3711220
Centrin2	Cheeseman Lab^82^	NA
GFP-Booster ATTO 488	Chromotek	gba488-100; RRID: AB_2827573
Ubiquitin (P4D1)	Santa Cruz Biotechnology	sc-8017; RRID: AB_628423
GFP nanobody	Purified in Cheeseman Lab	Addgene: 140442
PARP	Cell Signaling Technology	9532S; RRID: AB_659884
PABP1	Cell Signaling Technology	4992S; RRID: AB_10693595
RPS3	Cell Signaling Technology	D50G7; RRID: AB_10622028

Bacterial and virus strains		

MACH1 competent Cells	Thermo Fisher	C862003

Chemicals, peptides, and recombinant proteins		

Doxycycline	Sigma-Aldrich	Cat#D9891, CAS:24390-14-5
S-trityl-L-cysteine (STLC)	Sigma-Aldrich	Cat#164739, CAS:2799-07-7
Centrinone	Oegema lab^17^	NA
SiR tubulin	Cytoskeleton Inc.	Cat#: CY-SC002
Cycloheximide	Sigma-Aldrich	66-81-9
MG-132	Enzo Life Sciences	BML-PI102
Thymidine	Sigma-Aldrich	T9250
Suc-LLVY-AMC	R&D Systems, Inc.	S-280-05M
Paclitaxel (Taxol equivalent)	Life Technologies Corporation	P3456
TAK-243	Selleckchem	S8341
Puromycin	Gibco	A1113802
Propidium Iodide	Invitrogen	P3566
4′, 6-diamidino-2-phenylindole (DAPI)	EMD Millipore Corp	5.08741.0001

Critical commercial assays		

TMT Pro 16-plex Isobaric Labeling Reagent Set	Life Technologies Corporation	A44521
The Pierce High pH Reversed-Phase Peptide Fractionation Kit	Thermo Fisher	84868
LLC S-TRAP micro kit	Protifi	NC1828286

Deposited data		

Raw Mass Spectrometry Data	ProteomeXchange Consortium via the PRIDE (Proteomics Identifications Database)	PRIDE Database: PXD065357
Full Western Blot Images	Mendeley Data	Mendeley Data: https://doi.org/10.17632/k49vv5dh9p.1

Experimental models: Cell lines		

NIH-3T3	ATCC	ATCC-1568
HeLa	Cheeseman Lab	NA
HEK293T	Cheeseman Lab	NA
A549	Cheeseman Lab	NA
LLC-RK1 (Lilly Laboratories Cell - Rabbit Kidney 1; Rabbit)	ATCC	CCL106
MDCK (Madin-Darby Kidney Canine; Dog)	ATCC	CCL34
Doxycycline inducible: PSMD1 knockout (ggatcagcctatcaggaagg)	HeLa inducible Cas9,^25^ lentiviral transduction	cOM94
Doxycycline inducible: PSMD2 knockout (cctgtggaatgatagcagta)	HeLa inducible Cas9, lentiviral transduction	cOM160
Doxycycline inducible: PSMD3 knockout (ggccagcatcaaccacgaga)	HeLa inducible Cas9, lentiviral transduction	cOM96
Doxycycline inducible: PSMD4 knockout (ggcaagatcaccttctgcac)	HeLa inducible Cas9, lentiviral transduction	cOM104
Doxycycline inducible: PSMD6 knockout (tcgcgatgcaatgatggcaa)	HeLa inducible Cas9, lentiviral transduction	cOM161
Doxycycline inducible: PSMD7 knockout (tacagaagcgtacatttcag)	HeLa inducible Cas9, lentiviral transduction	cOM162
Doxycycline inducible: PSMD8 knockout (ccaatggagcatcctacgca)	HeLa inducible Cas9, lentiviral transduction	cOM99
Doxycycline inducible: PSMD11 knockout (ggaagcagctacagggcagg)	HeLa inducible Cas9, lentiviral transduction	cOM100
Doxycycline inducible: PSMD12 knockout (actctacgaatggttaccga)	HeLa inducible Cas9, lentiviral transduction	cOM103
Doxycycline inducible: PSMD13 knockout (tcgtgtagagctcctccaga)	HeLa inducible Cas9, lentiviral transduction	cOM163
Doxycycline inducible: PSMD14 knockout (ggagttccaatggaagttat)	HeLa inducible Cas9, lentiviral transduction	cOM152
Doxycycline inducible: PSMA1 knockout (ccatattggtatctcaattg)	HeLa inducible Cas9, lentiviral transduction	cOM169
Doxycycline inducible: PSMA6 knockout (gcttctaccaacatgtcccg)	HeLa inducible Cas9, lentiviral transduction	cOM112
Doxycycline inducible: PSMB1 knockout (agagactccttcaaggctgg)	HeLa inducible Cas9, lentiviral transduction	cOM111
Doxycycline inducible: PSMB5 knockout (cctgctaggcaccatggctg)	HeLa inducible Cas9, lentiviral transduction	cOM171
Doxycycline inducible: PSMB7 knockout (tggcacgaccatcgctgggg)	HeLa inducible Cas9, lentiviral transduction	cOM108
Doxycycline inducible: PSMC2 knockout (actgttgggtcaatcttagg)	HeLa inducible Cas9, lentiviral transduction	cOM165
Doxycycline inducible: PSMC4 knockout (gtgatgtacgcggacatcgg)	HeLa inducible Cas9, lentiviral transduction	cOM106
Doxycycline inducible: PSMC6 knockout (tgactacactaactatcatg)	HeLa inducible Cas9, lentiviral transduction	cOM167
Doxycycline inducible: KIF11 knockout (gaagttagtgtacgaactgg)	HeLa inducible Cas9, lentiviral transduction	cOM114
Doxycycline inducible: ADRM1 knockout (acgtgctgaagttcaaggca)	HeLa inducible Cas9, lentiviral transduction	cOM184
Doxycycline inducible: USP14 knockout (gaatacagatgaacctccaa)	HeLa inducible Cas9, lentiviral transduction	cOM172
Doxycycline inducible: UCH37 knockout (aagtacacaacagtttcgcc)	HeLa inducible Cas9, lentiviral transduction	cOM174
GFP-KIF11, N-terminally-tagged KIF11	cOM94, lentiviral transduction with pOM134	cOM151
GFP-KIF15	HeLa, transient transfection of GFP-KIF15 plasmid,^83^ with selection, clonal	cOM176.122, cOM176.129, cOM176.134
KIF11-GFP, C-terminally-tagged KIF11	HeLa, lentiviral transduction with pOM230	cOM216
GFP-PSMB4	cOM94, retroviral transduction with pJM18	cOM126
GFP-KIF11 (AA 1–361)	cOM94, lentiviral transduction with pOM148	cOM155
GFP-KIF11 (AA 762-end)	cOM94, lentiviral transduction with pOM150	cOM157
GFP-KIF11, KR mutant, (AA 762-end)	HeLa, lentiviral transduction with pOM231	cOM217

Oligonucleotides		

siRNA targeting PSMD1, pool: CAAAGGAUGCAGUACGGAA, AGACCAUACUGGAGUCGAA, GAUUGGAAGGCAUCGUAAA, CAUGGGAACUGCACGUCAA	Dharmacon	L-011363-01-0005
siRNA targeting PSMD1, individual: AGACCAUACUGGAGUCGAA	Dharmacon	CTM-1021344
siRNA targeting PSMD8, pool: GCUACUACUUUGAUUACAA, AAUUCUGGCCCGUGACAUA, GAGCUCAACUUCUUGCCAA, CAGUGUCCCUGGAGCAAUA	Dharmacon	L-017583-00-0005
siRNA targeting PSMB7, pool: CCGCAGGAAUGCCGUCUUG, GUAUCAAGGUUACAUUGGU, GGUCUAUAAGGAUGGCAUA, GAAGAUAAGUUUAGGCCAG	Dharmacon	L-006021-00-0005
siRNA targeting DYNC1H1, pool: GAUCAAACAUGACGGAAUU, CAGAACAUCUCACCGGAUA, GAAAUCAACUUGCCAGAUA, GCAAGAAUGUCGCUAAAUU	Dharmacon	L-006828-00-0005
siRNA targeting Lis1, pool: CAAUUAAGGUGUGGGAUUA, UGAACUAAAUCGAGCUAUA, GGAGUGCCGUUGAUUGUGU, UGACAAGACCCUACGCGUA	Dharmacon	L-010330-00-0005
siRNA control pool	Dharmacon	Cat#D-001810-10
CRISPR Knockout guides	This Manuscript	See Table S7
Primers used for TIDE analysis	IDT	See Table S7
Primers used for qPCR	IDT	See Table S7

Recombinant DNA		

Ub-R-GFP	Addgene	11939
pOM134, GFP-KIF11, N-terminal tag	This Manuscript	NA
pOM148, GFP-KIF11, Amino acids 1–361	This Manuscript	NA
pOM150, GFP-KIF11, Amino acids 762-end	This Manuscript	NA
pOM230, KIF11-GFP, C-terminal tag	This Manuscript	NA
pOM231, GFP-KIF11, Amino acids 762-end, Lysine to Arginine mutant	This Manuscript	NA
pJM18, GFP-PSMB4	Monda and Cheeseman^84^	NA
GFP-KIF15	Wadsworth Lab^83^	NA

Software and algorithms		

Fiji (ImageJ)	Schindelin et al.^85^	https://fiji.sc
GraphPad Prism V10.1.1	N/A	https://www.graphpad.com
ProteomeDiscoverer	ThermoFisher	https://www.thermofisher.com/order/catalog/product/OPTON-31014
FlowJo V10.8	N/A	https://www.flowjo.com
TIDE: Tracking of Indels by DEcomposition	Brinkman et al.^86^	https://tide.nki.nl
AlphaFold2	Jumper et al.^87^	https://alphafold.ebi.ac.uk/
Image Studio	LICORbio	https://www.licorbio.com/image-studio

## Highlights

- Loss of proteasome 19S lid subunits uniquely compromises bipolar spindle assembly
- Monopolar spindles from 19S lid loss are not due to reduced proteasome degradation
- 19S lid depletion induces ubiquitin-independent KIF11 degradation by the 20S core
- The 26S:20S proteasome ratio fluctuates throughout the cell cycle
