# Supplementary material for: 19S proteasome loss regulates mitotic spindle assembly through a ubiquitin-independent degradation mechanism
Source: Cell Rep. Author manuscript; Available in PMC 2025 Sep 13. (PMC12432587; doi:10.1016/j.celrep.2025.116041)
Supplement: 1 [file NIHMS2107259-supplement-1.pdf]

Cell Reports, Volume *44*

Supplemental information

19S proteasome loss regulates mitotic spindle assembly through a ubiquitin-independent degradation mechanism

Océane Marescal and Iain M. Cheeseman

Supplementary Figures

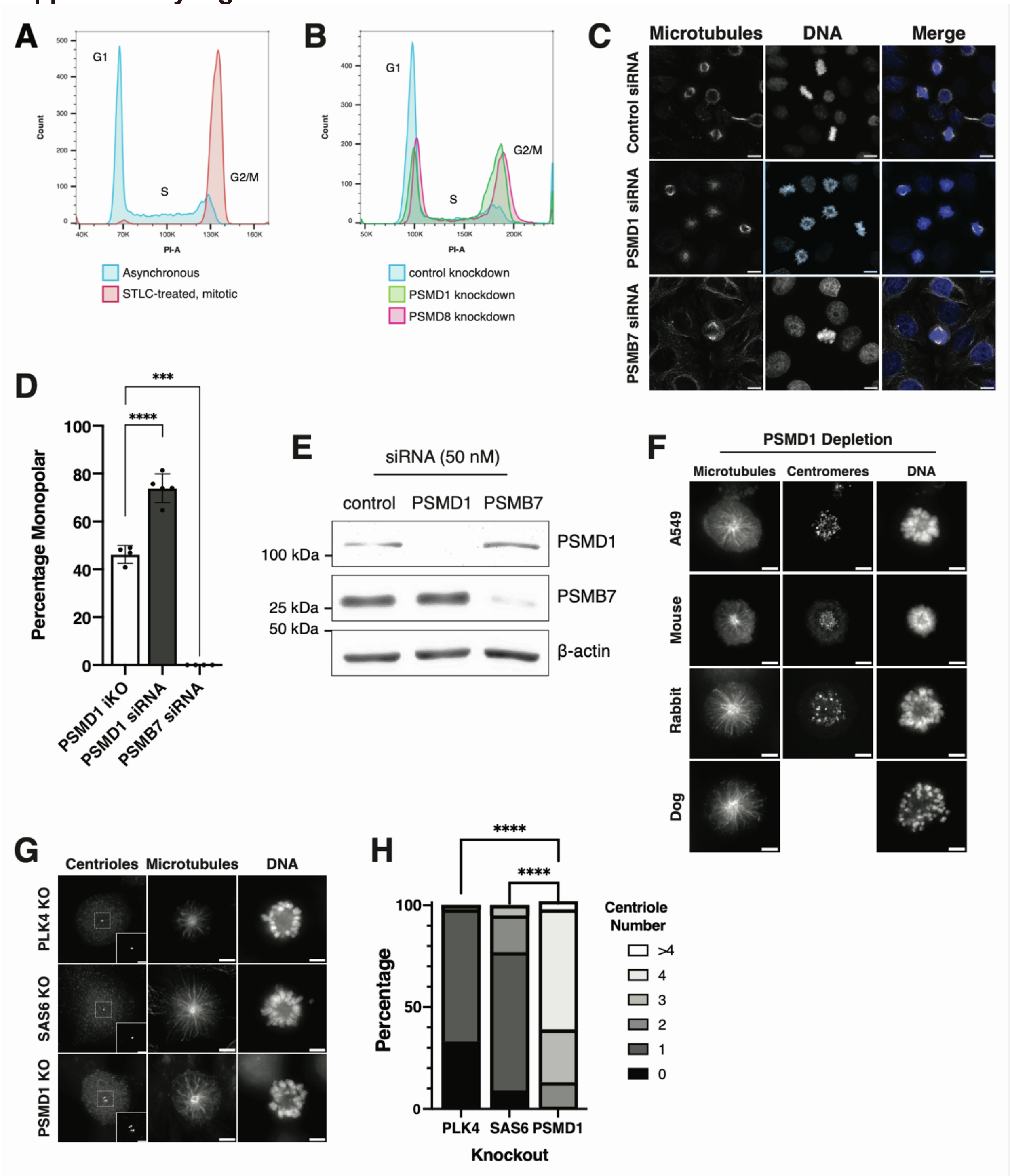

Figure S1: Alternative strategies for inducing proteasome subunit depletion and counting centriole numbers, Related to Figure 1. (A) Histogram showing the distribution of propidium iodide (PI) staining for asynchronous cells (blue) or cells arrested in mitosis using *S*-Trityl-L-cysteine (STLC, 10 µM, red) as measured by flow cytometry. G1, S, and G2/M cell cycle stages are labeled. **(B)** Histogram showing the distribution of propidium iodide (PI) staining for cells treated with 50nM control (blue), PSMD1 (green), or PSMD8 (pink) siRNAs as measured by flow cytometry. G1, S, and G2/M cell cycle stages are labeled. **(C)** Representative immunofluorescence images of asynchronous HeLa cells treated with 50nM control siRNAs,

HeLa cells treated with 50 nM PSMD1 siRNAs, and HeLa cells treated with 50 nM PSMB7 siRNAs. Scalebars = 10 µm. **(D)** Percentage of mitotic cells with monopolar spindles observed in inducible CRISPR-Cas9 PSMD1 knockout HeLa cells, HeLa cells treated with 50 nM PSMD1 siRNAs, and HeLa cells treated with 50 nM PSMB7 siRNAs. Bars represent mean ± standard deviation. P-values were calculated with two-tailed Welch's t-tests: **** represents $p < 0.0001$, *** represents $p = 0.0001$. **(E)** Western blot of cells treated with control, PSMD1, or PSMB7 siRNAs. Blot was incubated with PSMD1 or PSMB7 antibodies. β-actin is used as a loading control. **(F)** Representative immunofluorescence images of monopolar mitotic A549, mouse (3T3), Rabbit (LLC-RK1), and Dog (MDCK) cells resulting from PSMD1 depletion. A549 and 3T3 cells were transduced with lentivirus containing mCherry-expressing gene knockout plasmids for PSMD1 and sorted for mCherry before seeding on coverslips. LLC-RK1 and MDCK cells were treated with siRNAs targeting PSMD1. Scalebars = 5 µm. **(G)** Representative immunofluorescence images of monopolar mitotic cells from inducible CRISPR-Cas9 knockouts of PSMD1, PLK4, and SAS6. Cells were stained for centrioles with Centrin2 antibody. Regions enlarged in insets are outlined with dashed squares. Image brightness not identical. Scalebars for full-sized images = 5 µm. Scalebars for insets = 2 µm. **(H)** Quantification of centriole number in inducible CRISPR-Cas9 knockouts of PSMD1, PLK4, and SAS6. Bars show the percentage of cells in each centriole number category. Experiment was replicated 3 times, 31-60 cells were quantified for each condition for each replicate. P-values were calculated with chi-square tests. **** represents $p < 0.0001$.

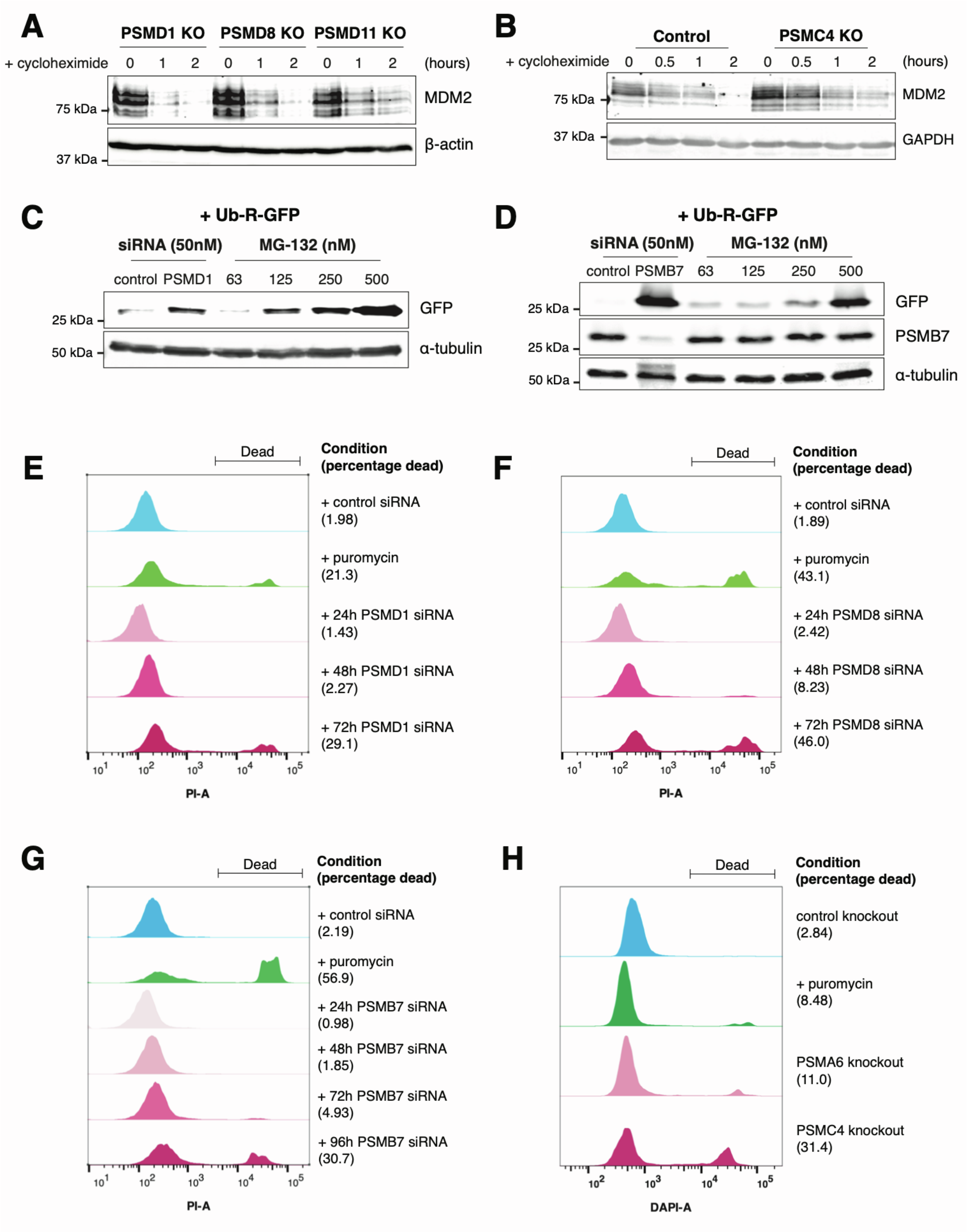

Figure S2: Assays for ubiquitin-mediated proteasome degradation activity and cell death, Related to Figure 2. (A) Western blot of inducible CRISPR-Cas9 PSMD1, PSMD8, and PSMD11 knockout cells treated with 50 μg/ml of cycloheximide for the indicated amount of time. Blot was incubated with MDM2

antibody. β-actin is used as a loading control. **(B)** Western blot of control and inducible CRISPR-Cas9 PSMC4 knockout cells treated with 50 μg/ml of cycloheximide for the indicated amount of time. Blot was incubated with MDM2 antibody. GAPDH is used as a loading control. **(C)** Western blot of Ub-R-GFP-transfected cells treated with different conditions: 50 nM control siRNAs, 50 nM PSMD1 siRNAs, and the indicated concentrations of MG-132. Cells were transfected with Ub-R-GFP plasmid 60 hours prior to collection. siRNAs were added 48 hours prior to collection, and MG-132 was added 24 hours prior to collection. Blot was incubated with GFP antibody. β-actin is used as a loading control. **(D)** Western blot of Ub-R-GFP-transfected cells treated with different conditions: 50 nM control siRNAs, 50 nM PSMB7 siRNAs, and the indicated concentrations of MG-132. siRNAs were added 72 hours prior to collection. Cells were transfected with Ub-R-GFP plasmid 48 hours prior to collection, and MG-132 was added 24 hours prior to collection. Blot was incubated with GFP antibody and PSMB7 antibody. α-tubulin is used as a loading control. **(E)** Histograms showing propidium iodide (PI) staining in cells treated with 50 nM control siRNAs, puromycin (1 μg/ml), and indicated times of 50 nM PSMD1 siRNAs. Dead cells stain positive for PI. Y-axis represents count. **(F)** Histograms showing propidium iodide (PI) staining in cells treated with 50 nM control siRNAs, puromycin (1 μg/ml), and indicated times of 50 nM PSMD8 siRNAs. Dead cells stain positive for PI. Y-axis represents count. **(G)** Histograms showing propidium iodide (PI) staining in cells treated with 50 nM control siRNAs, puromycin (1 μg/ml), and indicated times of 50 nM PSMB7 siRNAs. Dead cells stain positive for PI. Y-axis represents count. **(H)** Histograms showing DAPI (4', 6-diamidino-2-phenylindole) staining. HeLa cells were transduced with lentivirus containing the mCherry-expressing gene knockout plasmids for a cutting control (sgHS1), PSMA6, or PSMC4. mCherry-positive cells were then sorted by flow cytometry. Four days after virus transduction, cells were collect for cell death assay. Puromycin-treated cells (1 μg/ml) were used as a positive control. Dead cells stain positive for DAPI. Y-axis represents count.

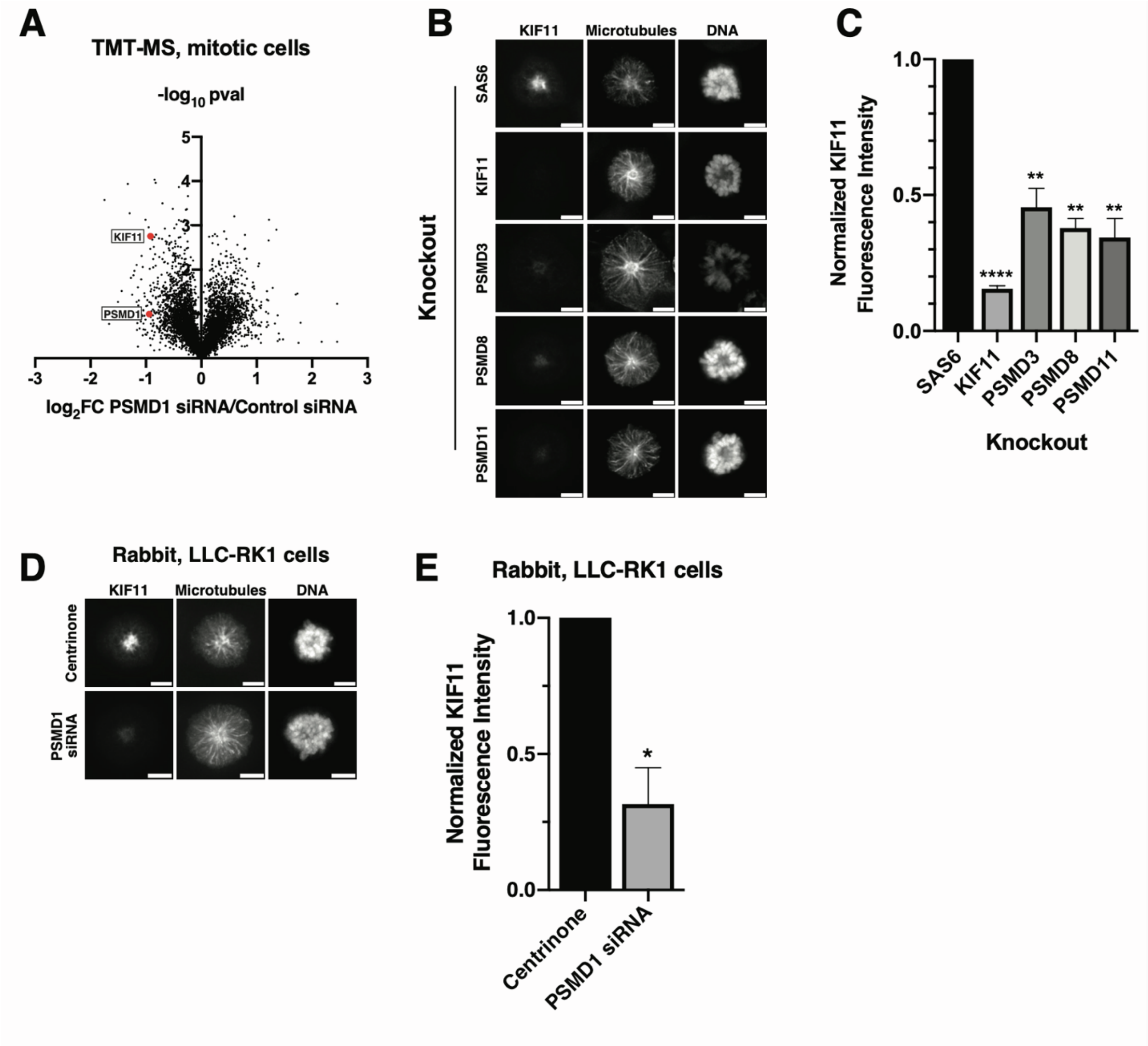

Figure S3: KIF11 is lost from mitotic cells upon PSMD1 depletion, Related to Figure 3. (A) Comparison of protein abundances in cells treated with 50 nM PSMD1 siRNAs and cells treated with 50 nM control siRNAs. Cells were transduced with lentivirus for a guide targeting control AAVS safe harbor locus. Then they were treated with siRNAs for either PSMD1 or control for 48 hours and arrested in mitosis with 1 μM taxol prior to harvesting. Mitotic cells were specifically isolated from interphase cells using mitotic shake off. Protein abundances were obtained using TMT-based (Tandem Mass Tag) quantitative mass spectrometry of three replicates for PSMD1 RNAi condition and two replicates for control RNAi condition. Volcano plot shows $\log_2$ of protein abundances in PSMD1 knockdown cells divided by protein abundances in control knockdown cells vs. $-\log_{10}$ of p-values. Values on the right of the plot represent proteins with increased abundance in PSMD1 knockdown cells and values on the left represent proteins with decreased abundance in PSMD1 knockdown cells. PSMD1 and KIF11 are highlighted in red. **(B)** Representative immunofluorescent images of monopolar mitotic cells showing KIF11 levels in inducible CRISPR-Cas9 knockouts of SAS6, KIF11, PSMD3, PSMD8, and PSMD11. Scalebar = 5 μm. **(C)** Quantification of KIF11 fluorescence intensity in monopolar mitotic cells in different conditions. Fluorescence intensity values were normalized to levels in control SAS6 knockout cells. Bars represent mean ± standard deviation. P-values were calculated with two-tailed Welch's t-tests comparing each condition to SAS6 knockout: $p < 0.0001$ for KIF11, $p = 0.0053$ for PSMD3, $p = 0.0011$ for PSMD8, and $p = 0.0037$ for PSMD11. **** represents $p < 0.0001$, ** represents $p < 0.01$. Experiment was replicated 3 times, 38-76 cells were quantified for each

condition for each replicate. **(D)** Representative immunofluorescent images of monopolar mitotic Rabbit LLC-RK1 cells showing KIF11 levels in either centrinone-treated cells (positive control) or PSMD1-siRNA treated cells. Scalebar = 5μm. **(E)** Bar graph showing KIF11 fluorescence intensity in centrinone-treated cells (positive control) or PSMD1 siRNA-treated cells. Values normalized to positive control. Bars represent mean ± standard deviation. P-values were calculated with two-tailed Welch's t-tests. * represents $p = 0.0124$ from 3 replicates. Between 39-93 cells were quantified for each condition for each replicate.

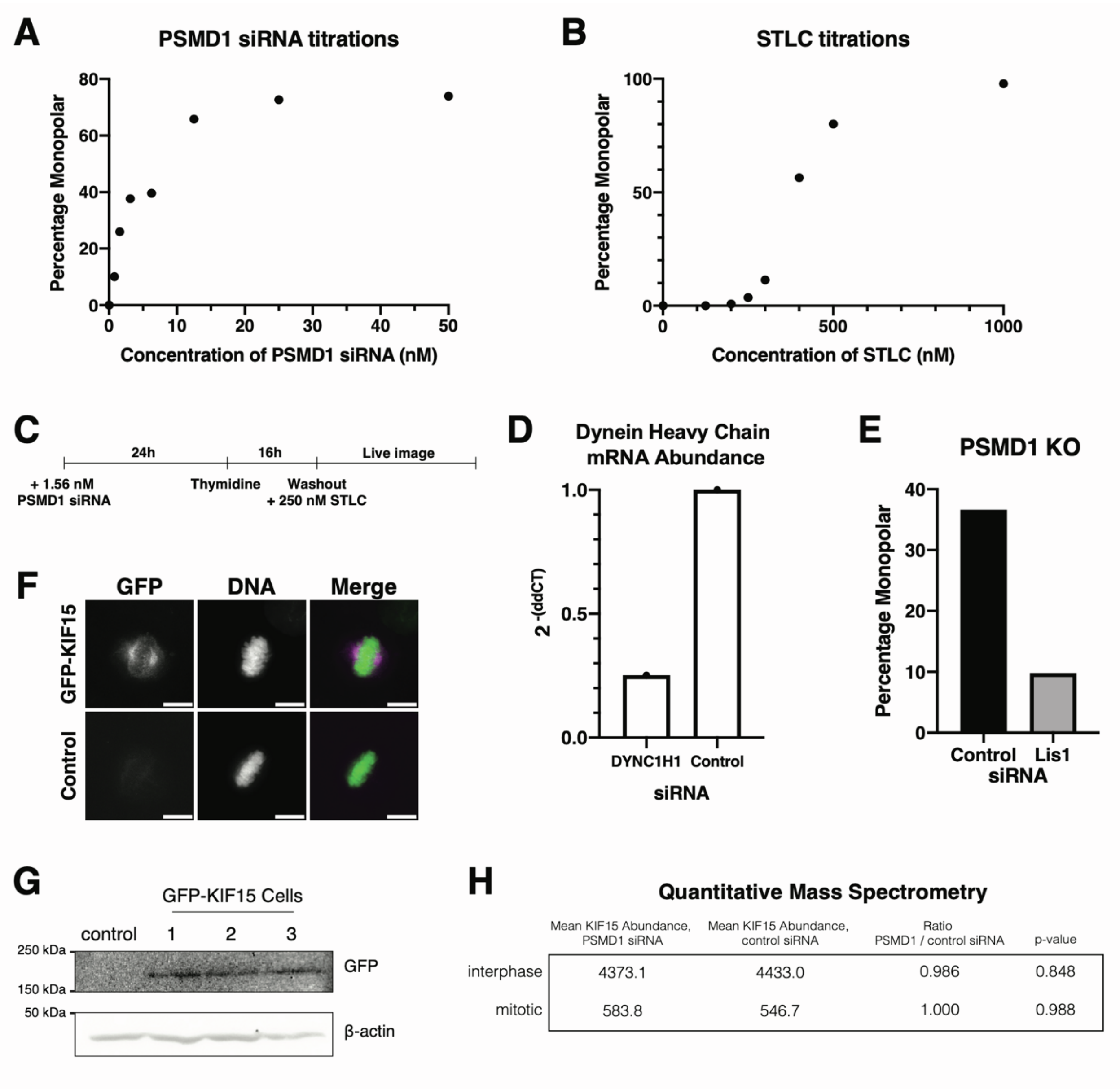

Figure S4: Forces acting on the mitotic spindle can be experimentally manipulated, Related to Figure 4. (A) Titration curve for PSMD1 siRNA titrations. PSMD1 siRNA concentrations were between 0 and 50 nM. The percentage of mitotic cells with monopolar spindles was quantified by immunofluorescence. **(B)** Titration curve for STLC titrations. STLC concentrations ranged from 0 to 1000 nM. The percentage of mitotic cells with monopolar spindles was quantified by immunofluorescence. **(C)** Diagram showing the experimental timeline for the STLC/PSMD1 siRNA synergy experiment shown in Figure 4A. **(D)** Graph showing the abundance of dynein heavy chain (DYNC1H1) mRNA as quantified by qPCR following treatment of cells with 50 nM of DYNC1H1 or control siRNAs. 3 technical replicates were conducted per condition for a single biological replicate. CT values for DYNC1H1 mRNA were normalized to those of GAPDH before normalization to control siRNA condition. **(E)** Quantification of live imaging experiment. Bar graph showing the percentage of cells entering mitosis with monopolar spindles in PSMD1 knockout cells treated with either 50 nM of control or Lis1 siRNA. Experiment was conducted 1 time and between 1300-1406 mitotic cells were counted for each condition. **(F)** Representative immunofluorescence images of GFP-KIF15 and control cells. Cells were stained with GFP booster. GFP-KIF15 localizes to the spindles. Scalebar = 10 μm **(G)** Western blot of GFP-KIF15 monoclonal cells lines and control parental cells blotted with GFP antibody. Size of KIF15 is 160 kDa. β-actin is used as a loading control. **(H)** Table showing

abundance of KIF15 in PSMD1 siRNA- vs. control siRNA-treated cells. Protein abundances were obtained using TMT-based (Tandem Mass Tag) quantitative mass spectrometry.

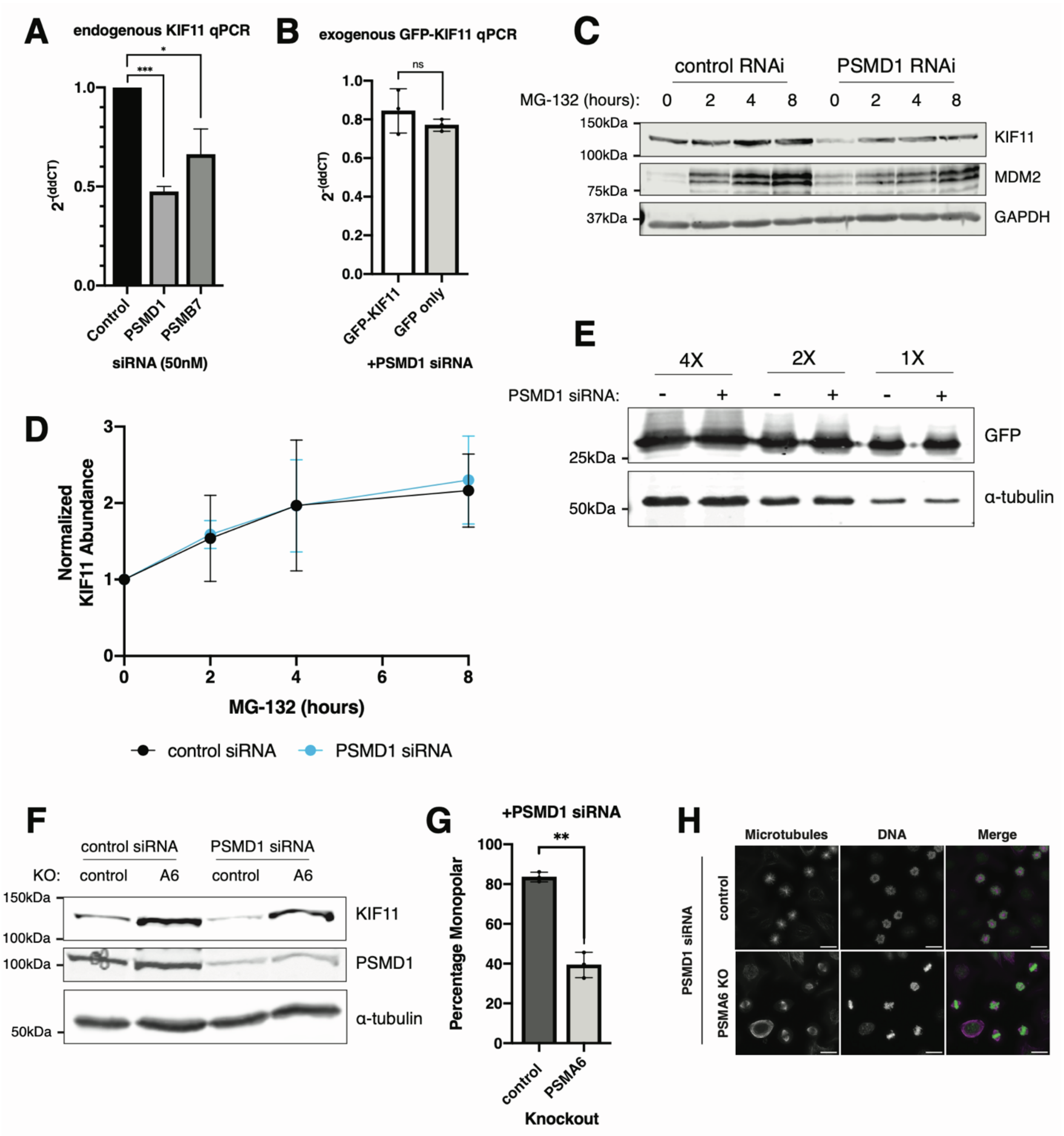

Figure S5: KIF11 mRNA levels in PSMD-depleted cells, Related to Figure 5. (A) Graph showing the abundance of endogenous KIF11 mRNA in different conditions as quantified by qPCR following treatment of cells with 50 nM control, PSMD1, or PSMB7 siRNAs. 3 technical replicates are shown per condition for a single biological replicate. Experiment was replicated two times with similar results. CT values for KIF11 mRNA were normalized to those of GAPDH before normalization to control siRNA condition. **(B)** Graph showing the relative decrease in the abundances for the indicated mRNAs following treatment of cell lines with PSMD1 siRNAs. GFP-KIF11 or GFP only cell lines were treated with 50 nM PSMD1 siRNAs for 48 hours prior to harvesting. 3 biological replicates with 3 averaged technical replicates each are shown per condition. CT values for KIF11 mRNA or GFP mRNA were normalized to those of GAPDH before normalization to respective control siRNA condition. **(C)** Western blot of cells treated with 50 nM control or PSMD1 siRNAs and the indicated times of MG-132 (10 μM). Blots were incubated in KIF11 or MDM2 antibody. GAPDH is used as a loading control. **(D)** Plot quantifying the increase in KIF11 levels after MG-

132 addition. KIF11 band intensities were divided by GAPDH band intensities and then normalized to the 0-hour MG-132 timepoint for each condition (control vs. PSMD1 siRNA). Bands from quantitative western blots were quantified using LICORbio Image Studio. Bars represent mean ± standard deviation. Values determined from 3 replicates. **(E)** Western blot of control GFP-expressing cells treated with 50 nM control (-) or PSMD1 (+) siRNAs. Blot was incubated in GFP antibody. The same samples were loaded at different concentrations (4X, 2X, or 1X). α-tubulin is used as a loading control. **(F)** Western blot of control cells or PSMA6 knockout cells treated with either 50 nM control or PSMD1 siRNAs. Knockouts were generated by transducing HeLa cells with lentivirus containing the mCherry-expressing gene knockout plasmids for PSMA6. Cells were then sorted for mCherry positive cells by FACS. Blot was incubated with KIF11 and PSMD1 antibodies. α-tubulin is used as a loading control. **(G)** Quantification of immunofluorescence experiment from PSMA6 knockout cells. Bar graph shows the percentage of mitotic cells with monopolar spindles for control or PSMA6 knockout cells treated with 12.5 nM PSMD1. P-value was calculated with two-tailed Welch's t-tests, $p = 0.0031$. ** represents $p < 0.01$. Experiment was conducted 3 times. **(H)** Representative immunofluorescence images from Figure S5G. Control (top) or PSMA6 knockout cells (bottom) were treated with 12.5 nM PSMD1 siRNAs. Scalebar = 20 μm.

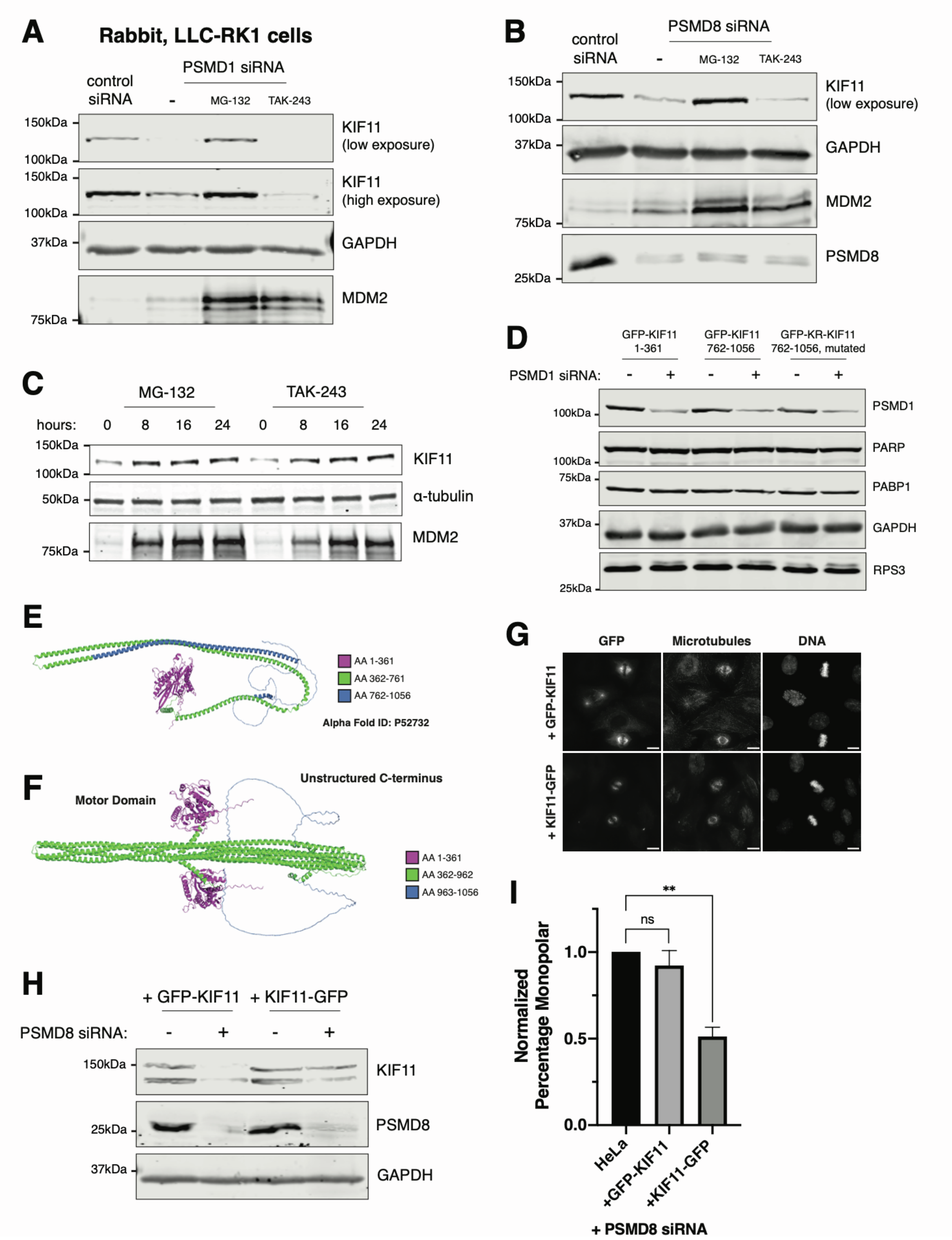

Figure S6: KIF11 degradation is ubiquitin-independent and depends on its C-terminus, Related to Figure 6. (A) Western blots of rabbit LLC-RK1 cells treated with 50 nM of the indicated siRNAs and the

indicated compounds. MG-132 was used at 10 µM and TAK-243 at 300 nM. siRNAs were added 48 hours prior to collection and compounds were added 24 hours prior to collection. Blots were incubated with KIF11 and MDM2 antibodies. GAPDH is used as a loading control. Separate blots were used with the same samples and same amounts loaded. One blot was incubated with KIF11 and GAPDH antibodies and the other with MDM2 antibody. **(B)** Western blots of HeLa cells treated with 50 nM of the indicated siRNAs and the indicated compounds. MG-132 was used at 10 µM and TAK-243 at 300 nM. siRNAs were added 48 hours prior to collection and compounds were added 24 hours prior to collection. Blots were incubated with KIF11, PSMD8, and MDM2 antibodies. GAPDH is used as a loading control. Separate blots were used with the same samples and same amounts loaded. One blot was incubated with KIF11, PSMD8, and GAPDH antibodies and the other with MDM2 antibody. **(C)** Western blot of control cells treated with MG-132 or TAK-243 for the indicated amount of time. Blots were incubated with KIF11 or MDM2 antibodies. α-tubulin is used as a loading control. Separate blots were used with the same samples and same amounts loaded. One blot was incubated with KIF11 and α-tubulin antibodies and the other with MDM2 antibody. **(D)** Western blots of KIF11 construct-expressing cell lines treated with 50 nM of control (-) or PSMD1 (+) siRNAs. Blots were incubated with PSMD1 antibody and negative control antibodies: PARP, PABP1, GAPDH, and RPS3. Separate blots were used with the same samples and same amounts loaded. One blot was incubated with GAPDH and PARP antibodies and the other with PSMD1, PABP1, and RPS3 antibodies. **(E)** Alpha-fold prediction of full-length Homo sapiens KIF11 structure. AF-P52732-F1-v4. **(F)** Alpha-fold prediction of full-length dimer of Homo sapiens KIF11 structure. **(G)** Representative images of GFP-KIF11-expressing cell lines. Cell lines shown characteristic KIF11 localization at the spindles. Scale bars = 10 µm. **(H)** Western blot of cell line expressing N-terminally-tagged KIF11-GFP construct or cell line expressing C-terminally-tagged KIF11-GFP construct. Cells were treated with either 50 nM control (-) or PSMD8 (+) siRNAs. Blot was incubated with KIF11 and PSMD8 antibodies. GAPDH is used as a loading control. **(I)** Quantification of immunofluorescence experiment for cells expressing KIF11 constructs. Bar graph shows the percentage of mitotic cells with monopolar spindles for parental HeLa cells, a cell line expressing N-terminally-tagged GFP-KIF11, and a cell line expressing C-terminally-tagged KIF11-GFP treated with 6.25 nM PSMD8 siRNAs. Values were normalized to mean of control HeLa cells. ** indicates $p < 0.01$, ns = not significant. 724-1117 were counted for each replicate for each condition.

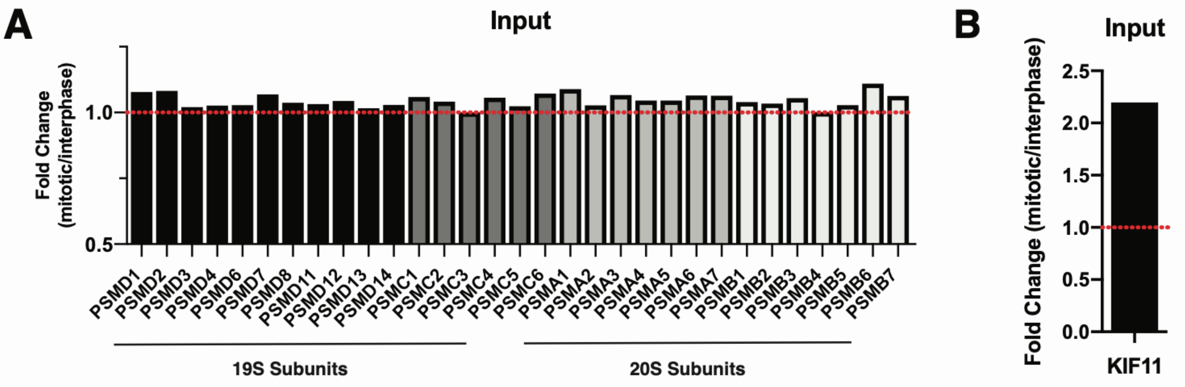

C Structural Conservation of KIF11 Disordered C-terminus

Organism	Disordered Residues	Alpha Fold ID
Homo sapiens (Human)	916-1056	AF-P52732-F1-v4
Pan troglodytes (Chimpanzee)	917-1041	AF-A0A2J8PE77-F1-v4
Mus musculus (Mouse)	916-1052	AF-Q6P9P6-F1-v4
Oryctolagus cuniculus (Rabbit)	955-1091	AF-A0A5F9CZS7-F1-v4
Bos taurus (Cattle)	917-1056	AF-E1BF29-F1-v4
Physeter macrocephalus (Sperm Whale)	919-1056	AF-A0A2Y9SIE7-F1-v4
Xenopus laevis (Frog)	927-1067	AF-Q91783-F1-v4
Drosophila melanogaster (Fly)	924-1066	AF-P46863-F1-v4
Saccharomyces cerevisiae (Yeast)	929-1000	AF-P27895-F1-v4

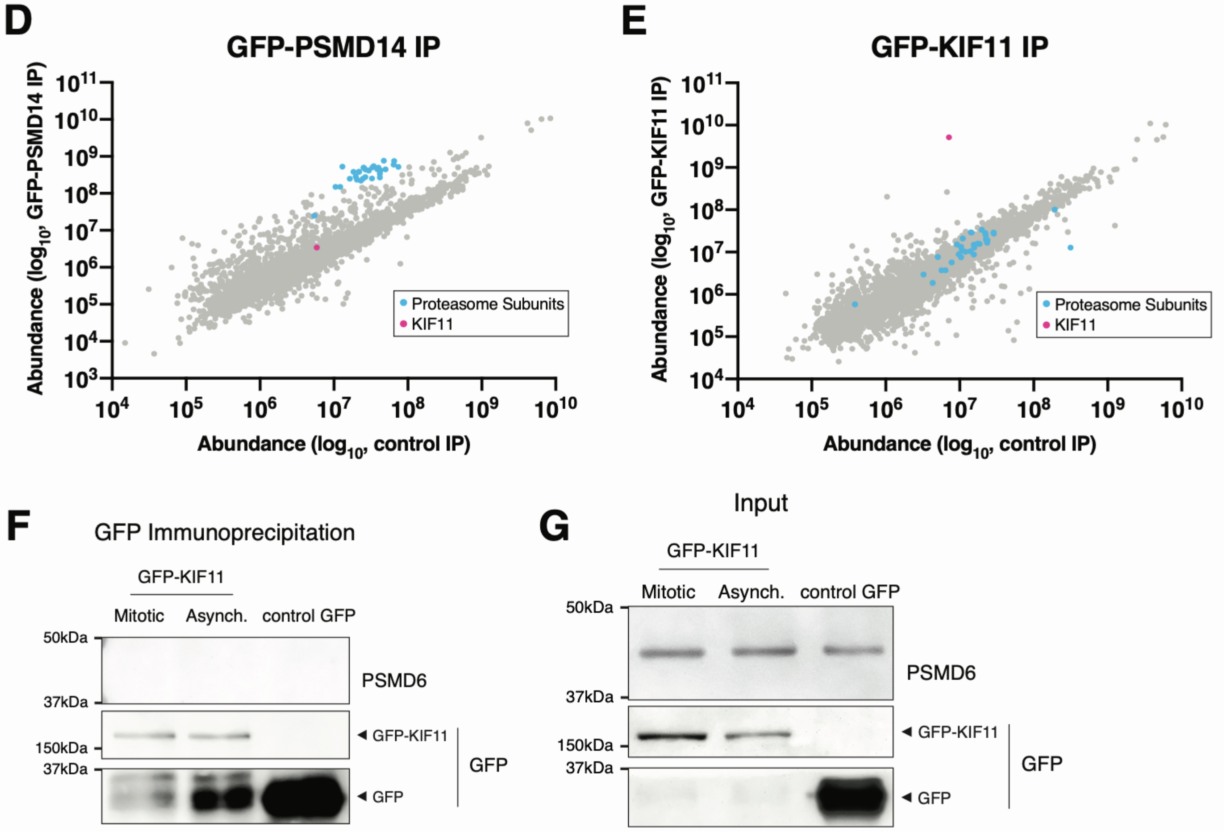

Figure S7: Protein abundances in the cell cycle, conservation of KIF11's unstructured C-terminus, and KIF11 or 19S immunoprecipitations, Related to Figure 6 and to Discussion. (A) Bar graph showing fold change of input protein abundances in mitotic (STLC-arrested) compared to asynchronous

cells as measured by Tandem Mass Tag (TMT) quantitative mass spectrometry from experiment in Figure 6G. Samples were collected prior to GFP immunoprecipitation. Bar graph shows the abundance ratios of mitotic/asynchronous values for proteasome 19S and 20S subunits. **(B)** Bar graph showing fold change of input protein abundances in mitotic (STLC-arrested) compared to asynchronous cells as measured by Tandem Mass Tag (TMT) quantitative mass spectrometry. Samples were collected prior to GFP immunoprecipitation. Bar graph shows the abundance ratios of mitotic/asynchronous values for KIF11. **(C)** Table showing the conservation of KIF11 unstructured C-terminus across different species. **(D)** Plot showing mass spectrometry results of GFP immunoprecipitations (IP) from GFP control (x-axis) and GFP-PSMD14 (y-axis) cell lines. IP was performed on mitotic cells. Proteasome subunits are labeled in blue and KIF11 is labeled in pink. GFP-PSMD14 co-immunoprecipitates all proteasome subunits above control levels, but not KIF11. Data corresponds to Supplementary Table 6. **(E)** Plot showing mass spectrometry results of GFP immunoprecipitations (IP) from GFP control (x-axis) and GFP-KIF11 (y-axis) cell lines. IP was performed on asynchronous cells. Proteasome subunits are labeled in blue and KIF11 is labeled in pink. KIF11 does not co-immunoprecipitate proteasome subunits. Data corresponds to Supplementary Table 6. **(F)** Western blot showing co-immunoprecipitation results. GFP immunoprecipitation was performed on a GFP-KIF11 cell line in mitotic and asynchronous cells, as well as asynchronous control GFP cells. Blot was incubated in PSMD6 and GFP antibodies. GFP-KIF11 and GFP bands are indicated. **(G)** Western blot showing input samples from immunoprecipitation performed in Figure S7F. Blot was incubated in PSMD6 and GFP antibodies. GFP-KIF11 and GFP bands are indicated.

Table S1: Percentage editing of "one-shot" CRISPR-Cas9 knockouts
Table showing the genome cutting efficiency of proteasome subunit CRISPR-Cas9 knockouts generated by lentiviral transduction of mCherry-containing gene knockout plasmids, as measured by TIDE (Tracking of Indels by Decomposition).

**Percentage Editing of "one-shot" CRISPR-Cas9 Knockouts**	
**Subunit**	**Percentage Editing**
PSMD1	84.7
PSMD8	78.6
PSMD11	94.0
PSMA6	65.6
PSMC4	52.6

Table S7: Oligonucleotide sequences. *Top*: CRISPR-Cas9 knockout guide sequences. *Middle*: Primer sequences used to amplify genomic DNA fragments by PCR from knockout cells for analysis of genome cutting efficiency by TIDE (Tracking of Indels by Decomposition). *Bottom*: Primer sequences used for qPCR.

**CRISPR knockout guides**		
**Gene**	**Guide sequence**	
PSMD1	ggatcagcctatcaggaagg	
PSMD2	cctgtggaatgatagcagta	
PSMD3	ggccagcatcaaccacgaga	
PSMD4	ggcaagatcaccttctgcac	
PSMD6	tcgcgatgcaatgatggcaa	
PSMD7	tacagaagcgtacatttcag	
PSMD8	ccaatggagcatcctacgca	
PSMD11	ggaagcagctacagggcagg	
PSMD12	actctacgaatggttaccga	
PSMD13	tcgtgtagagctcctccaga	
PSMD14	ggagttccaatggaagttat	
PSMA1	ccatattggtatctcaattg	
PSMA6	gcttctaccaacatgtcccg	
PSMB1	agagactccttcaaggctgg	
PSMB5	cctgctaggcaccatggctg	
PSMB7	tggcacgaccatcgctgggg	
PSMC2	actgttgggtcaatcttagg	
PSMC4	gtgatgtacgcggacatcgg	
PSMC6	tgactacactaactatcatg	
ADRM1	acgtgctgaagttcaaggca	
USP14	gaatacagatgaacctccaa	
UCH37	aagtacacaacagtttcgcc	
KIF11	gaagttagtgtacgaactgg	

SAS6	ccaccaactagtcccgttgc	
PLK4	agagctgagtccattcacac	
**TIDE primer sequences**		
**Gene**	**Forward**	**Reverse**
PSMD1	gtgaacattctggggaaaaggcag	gcccagccctggcttcaatc
PSMD2	ccttggggctgtcacaaggg	cccagagcctgcgtcagg
PSMD3	gcctggtggcaggttcctgtaatc	gcaagcaagacaggtaacacctcc
PSMD6	cgtgggcagactttgacttgag	ctgaaaagcctatccctgggcag
PSMD7	ggctgtgttgactctaatgtccttg	cagcgtctcaactgtcgcac
PSMD8	ccacagggaccaagctgacc	gctgggatgagcaggcacag
PSMD11	cgttgggtcagtttcttaagtggg	cagggtctagtctgaacttgtggg
PSMD12	ccgtgataagatacccaattcccag	ccgtttcgccttttgcctctg
PSMD13	gagcatttccggcagccatc	ccgtgcaaccagctgcag
PSMD14	gatgctcctgcagtggacacag	caggtaatttgtatggagtacggtgc
PSMA1	caaggttcagccacagttggtc	ccctgcagtatccagtattgagc
PSMA6	ctcactccaccacccccttagg	ggcttcgagtgcctgtgtgg
PSMB1	ggtttctgtaggacacgtctgcc	caccatctctctctgtgttctccc
PSMB5	gatgtgctgggtttggattgatgc	cacctcctgggttcaagcaattctc
PSMB7	gcagtcgctcctgaaagacgg	gctccaccagttggaggcttc
PSMC2	ctcccctccctcctcaaagg	cagccaccaacatcactgtatgtgac
PSMC4	gcctcagtggccctccacaag	cattaactgctcactatcaccggg
PSMC6	gctgcctcaagctttgtgtgttag	cctctcttaattcccggatctgttctg
ADRM1	ctggcttctctgccttgtggag	ctctagacccaaaagacgctctgc
**qPCR primer sequences**		
**Gene**	**Forward**	**Reverse**
DYNC1H1	cgctcaaagaggacgtcgg	cggagctgagaagacacggg
KIF11 endogenous	ggaagaggatcccttggctgg	cactcctctcttgttacggggatc
GFP only	cagtttagaggtacttgtgctgacctg	cgaggtcaggagatcgagaccatc
GFP-KIF11	ggcatggacgagctgtacaagtc	gaatttggctgcgacgccatc
GAPDH	tcggagtcaacggatttggt	ttcccgttctcagccttgac

Table S8: List of antibodies used and their dilutions.

Primaries	company	dilution	Identifier	Animal
α-tubulin	Sigma-Aldrich	1 to 1000 (IF), 1 to 4000 (WB)	CAT#: T9026-.5ML; RRID: AB_477593	mouse monoclonal
ACA	Antibodies, Inc.	1 to 100 (IF)	CAT#: 15-234-0001	Human
PSMD1	Abcam	1 to 10000 (WB)	ab140682, Lot: GR285203-34	Rabbit polyclonal
PSMB7	Proteintech	1 to 1000 (WB)	Catalog Number: 30283-I-AP	Rabbit polyclonal
PSMD8	Proteintech	1:1000 (WB)	Catalog Number: 27504-I-AP	Rabbit polyclonal
PSMD6 (Proteasome 19S Rpn7)	Enzo	1:1000 (WB)	Catalog Number: BML-PW8225-0100	Rabbit polyclonal
β-actin (HRP conjugated)	Santa Cruz Biotechnology	1 to 5000 (WB)	sc-47778 HRP, Lot # G2523	mouse monoclonal
MDM2	Cell Signaling Technology	1 to 1000 (WB)	D1V2Z, Lot: 4	Rabbit monoclonal
GFP	Roche	1 to 5000 (WB)	Catalog Number: 11814460001	mouse monoclonal
GAPDH	Santa Cruz Biotechnology	1 to 5000 (WB)	sc-47724, Lot # H2521	mouse monoclonal
Eg5/KIF11	Abcam	1:500 (IF), 1 to 10000 (WB)	ab181981, Lot: 1089778-1	Rabbit monoclonal
centrin2	Cheeseman Lab[83]	1:12000 (IF)	NA	Rabbit polyclonal
GFP-Booster ATTO 488	Chromotek	1:200 (IF)	gba488-100, lot: 90401001AT1-06	VHH nanobody
Ubiquitin (P4D1)	Santa Cruz Biotechnology	1:200 (WB)	sc-8017, Lot # F2821	mouse monoclonal
GFP nanobody	Cheeseman Lab	50 µg/300µl (IP)	NA	VHH nanobody
PARP	Cell Signaling Technology	1:1000 (WB)	9532S	Rabbit monoclonal
PABP1	Cell Signaling Technology	1:1000 (WB)	4992S	Rabbit polyclonal
RPS3	Cell Signaling Technology	1:1000 (WB)	D50G7, Lot: 2	Rabbit monoclonal
